# Using intervention mapping to develop an occupational advice intervention to aid return to work following hip and knee replacement in the United Kingdom

**DOI:** 10.1186/s12913-020-05375-3

**Published:** 2020-06-09

**Authors:** Carol Coole, Paul Baker, Catriona McDaid, Avril Drummond

**Affiliations:** 1 School of Health Sciences, University of Nottingham, Nottingham, NG7 2HA England; 2 Medical School, Queens Medical Centre, University of Nottingham, Nottingham, NG7 2HA England; 3 The James Cook University Hospital, South Tees Hospitals NHS Foundation Trust, Middlesbrough, TS4 3BW England; 4 Department of Health Sciences, University of York, Seebohm Rowntree Building, Heslington York, YO10 5DD England

**Keywords:** Intervention mapping, Return to Work., Occupational advice., Arthroplasty., Hip, Knee.

## Abstract

**Background:**

There are increasing numbers of total hip replacements (THR) and total knee replacements (TKR) being performed in patients of working age. Providing patients undergoing TKR and THR with return to work advice might facilitate return to work. The aim of this paper is to report on the process used to systematically develop an occupational advice intervention to be delivered in hospital for those undergoing arthroplasty.

**Methods:**

The six-step Intervention Mapping (IM) approach to development, implementation and evaluation of a theory and evidence-based interventions was followed. This paper reports on the development of the intervention covered by steps 1 to 4 of the IM process. Steps 1–3 gathered data on current practice and barriers to change using a mixed methods approach (cohort study of patients undergoing THR or TKR, stakeholder interviews, survey of practice, evidence synthesis) and provided a theoretical framework for intervention development. Step 4 used information from steps 1–3 in combination with a Delphi consensus process to develop the intervention and the associated tools and materials to facilitate its delivery.

**Results:**

The final intervention identified included a number of core principles including: early patient identification; delivery of key information to patients and their employers; assessment and support by a member of the orthopaedic team; procedures for escalation based on patient need; mechanisms to support communication; and training and support for the clinical teams delivering care. A total of 13 patient and 20 staff performance objectives as delivery requirements, were supported by a range of tools, roles and training resources. The intervention addressed outcomes based at the individual and interpersonal levels of the ecological model.

**Conclusions:**

Following the IM approach resulted in a structured and justified occupational intervention for delivery in secondary care for patients undergoing total hip and knee replacement. The feasibility of the intervention will subsequently be tested alongside further investigation to establish its effectiveness and cost-effectiveness.

## Background

Hip and knee osteoarthritis is associated with reduced work participation [1] and productivity [2] and impacts on likelihood of employment, household income and sickness absence [3]. The direct and indirect costs of work related musculoskeletal disorders are borne by the individual, employers and society [4, 5]. Loss of employment is associated with a reduction in physical function, increased anxiety and depression and increased risk of mortality [6, 7]. Consequently, timely, sustained return to work after a period of sickness absence has potential health as well as socioeconomic benefits.

Lower limb joint replacements are effective and cost-effective treatments that relieve pain, restore physical function and improve health related quality of life for patients with hip and knee arthritis [8–11]. In the majority of western healthcare systems between 150 to 300 per 100,000 of the population undergo a total hip replacement (THR) [12, 13] and between 150 to 250 per 100,000 of the population undergo a total knee replacement (TKR) [12, 13] annually. There has been a steady rise in the number of hip and knee replacements performed each year since 2000 [12, 14] and these numbers are projected to increase significantly over the next 15 years [15, 16].

Recent changes to the state pension age, combined with an ageing workforce, have resulted in a steady increase in the numbers of hip and knee replacements being performed in United Kingdom (UK) patients of working age over the last decade [14]. These changes are also reflected in data from North America which suggest that over half of all hip and knee replacement procedures will be performed in patients aged under 65 years by 2030 [15]. International estimates suggest that between 15 and 45% of patients undergoing either hip or knee replacements are of working age [17, 18].

In the UK, less than two-thirds of large employers (250+ employees) and less than one half of medium-sized employers (51–249 employees) have access to occupational health [19]. There is also variation in the composition and support provided by workplace occupational health services ranging from in-house departments staffed by a full-time medical team to ad-hoc services provided by a single-handed occupational health nurse. National survey data demonstrates that there is substantial variation in the timing, content and delivery of return to work advice for patients undergoing hip and knee replacement with the majority receiving no advice or support from their healthcare team [20]. Thus many working people have little or no support to enable their return to work and are reliant on their own resources to manage their health conditions in the workplace. Providing patients undergoing TKR and THR with return to work advice and support from within NHS secondary care might enable them to return to work safely and effectively. The OPAL study (Occupational Advice for Patients undergoing Arthroplasty of the Lower Limb) [21] was therefore designed to develop an individualised occupational advice intervention that could be offered to any patient undergoing hip or knee replacement irrespective of their access to other occupational services. This intervention was intended to fit easily alongside routine care and take in to account the practicalities of implementation and delivery within the UK NHS healthcare setting.

The aim of this paper is to provide an overview of the intervention mapping process used in the OPAL study to systematically develop an occupational advice intervention.

## Methods

Return to work with, or following, a health problem is a complex intervention involving many potential stakeholders and levels of influence [22]. As such, a methodology was required that would address this challenge: it was believed that Intervention Mapping (IM) was most appropriate. IM is a framework for developing effective theory- and evidence-based behaviour change interventions [23, 24]. IM was developed for, and is widely used in health promotion, but has also been used in rehabilitation, for example in the management of osteoarthritis and back pain [25] and stroke [26] as well as in work disability prevention [27]. The IM framework was first used in work disability prevention in 2007. Interventions developed using this methodology have included self-management at work of chronic diseases [28] and upper limb conditions [29]. Only one study has focused on return to work following elective surgery [30]. The main characteristics of the IM protocol are to consider the individual within all the different levels of their environment, and to make explicit use of theories when defining the problem, the intended changes, and how these changes will be achieved. In this way, IM has the potential to prevent both theory and execution failures when developing and implementing return to work interventions, with better chances of demonstrating effectiveness.

The OPAL research team, with representation from orthopaedic surgeons, patients, therapists, occupational health and occupational psychology professionals, formed a participatory planning group. The team included one researcher trained in Intervention Mapping. The team met regularly throughout the study, either face-to-face or virtually. The activities of the team were also monitored by an independent committee comprising an orthopaedic surgeon, a trial methodologist and physiotherapist, a patient, a General Practitioner (GP), and a commissioner/retired GP.

The team followed the six-step IM approach to theory, evidence based development and implementation of interventions. This paper reports on the development of the intervention in steps 1 to 4 of the IM process. Steps 1–3 gathered data on current practice and barriers to change using a mixed methods approach (cohort study of patients undergoing THR or TKR, stakeholder interviews, survey of practice, evidence synthesis) and provided a theoretical framework for intervention development. Step 4 used information from steps 1–3 in combination with a Delphi consensus process to develop the intervention and the associated tools and materials to facilitate its delivery.

### Step 1

The team conducted a needs assessment to create a logic model of the problem. The needs assessment comprised four elements:

1. A rapid evidence review (PROSPERO registration number CRD42016045235 (Date registered August 2016)) of existing quantitative and qualitative evidence on occupational advice interventions for people undergoing any type of elective surgery or with chronic musculoskeletal problems and a mapping of currently used outcome measures to assess effectiveness. The review included 4 studies of return to work (RTW) interventions relating to elective surgical procedures and 17 systematic reviews of RTW interventions in the wider musculoskeletal literature. Key intervention components effective across previous RTW interventions were identified, including job accommodations, contact with employers, educational programmes and multidisciplinary involvement.
2. A prospective cohort study of patients undergoing total hip or knee replacement from four National Health Service (NHS) trusts was conducted between November 2016 and August 2017. Patients were eligible for inclusion if they were in paid (full-time, part-time, self-employed) or unpaid (volunteers or unpaid carers) work in the 6 months prior to surgery and intended to return to work after surgery. A total of 765 unselected hip and knee patients were screened, of which 196 (25.6%) were eligible for inclusion and 154 provided written consent and baseline data. Questionnaire assessments prior to and following surgery (8, 16, 24 weeks) provided information on patient characteristics, employment details (job roles, hours worked, employer characteristics), workplace assessments, functional outcomes, health utility measures, expectations of recovery, and rates and timing of return to work after surgery.
3. A web-based survey of current practice in the delivery of occupational advice with hospital orthopaedic teams (HOTs) involved in the treatment of hip and knee replacement in the UK. The survey asked respondents about the current delivery, timing and content of RTW advice within the UK health service. The survey was conducted between July 2017 and August 2017 and was disseminated via the National Joint Registry (NJR) for England, Wales and Northern Ireland clinician leads in 149 individual health trusts, the NJR eBulletin and the Scottish Committee for Orthopaedics and Trauma (SCOT). Responses were received from a total of 152 participants from 59 different public and private health providers [20].
4. A qualitative study of different stakeholder groups engaged in the RTW process. This element obtained information about current care related to RTW support, barriers preventing return to work, how these might be overcome, and how to translate this into an occupational advice intervention. Semi-structured interviews were conducted with a purposive sample of 45 patients undergoing THR or TKR at 3 NHS trusts, 25 workplace representatives (managers, human resources, occupational health, and colleagues), 16 GPs and 24 hospital orthopaedic staff between October 2016 and September 2017. Data were analysed using a framework approach that identified key themes relating to the RTW process [31–33].

The cohort questionnaires, the survey of current practice and the interview guides used for these interviews were developed specifically for the OPAL study. Examples of the cohort questionnaires and interview schedules are provided in Additional files 1 and 2.

The team summarised the key information developed from IM Step 1 in the context of the wider OPAL study aims [21], based on the PICO format (Population, Intervention, Comparator, Outcome) [34] (Additional file 3). Examples are shown in Table 1.

**Table 1:** Examples of information developed from Step 1

PICO	Information	Source
Population: Patients in work (to include full-time, part-time, self-employed, carers and volunteers) prior to hip or knee replacement surgery that intend to return to work after surgery	A substantial proportion (up to 25% of patients) are in work prior to surgery including some past state pension age	Cohort study
Intervention: an occupational advice intervention	Employers are reliant on employee feedback and not necessarily aware of the information patients receive	Interview study
Comparator: advice currently provided to RTW patients	The delivery of occupational advice is not generally seen as the role of, or a priority for, the orthopaedic team	Interviews and survey
Outcome: measurement of RTW	There is no standardised method of measuring RTW	Rapid evidence review

**Fig. 1:**
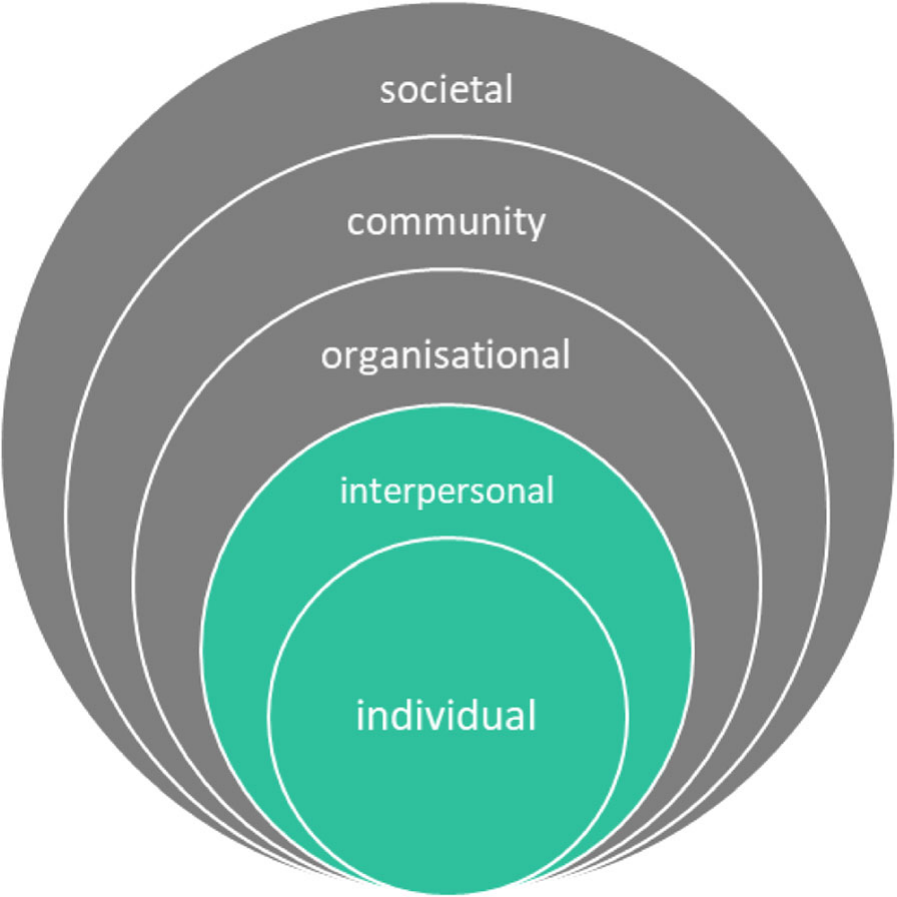
Ecological model. Adapted from Bronfenbrenner (1977). Toward an experimental ecology of human development. American psychologist 32(7) 513–531

Having explored the issues relating to return to work for people undergoing hip and knee replacement, the next task was to create a logic model to better understand the problem. Failing to return to work when fit to do some work, or returning to work too soon which may impede full recovery, potentially increases the risk of patients not achieving sustained return to their usual/expected work following THR/TKR. The theory- and evidence- based factors causally related to these patient behaviours include patients’ knowledge and beliefs about the recovery process in relation to return to work; their attitudes to and expectations of return to work; matters related to financial/job security; and their confidence in managing their recovery and RTW. Following the ecological model (Fig. 1), several environmental factors were identified that could directly or indirectly influence these patient behaviours.

**Fig. 2:**
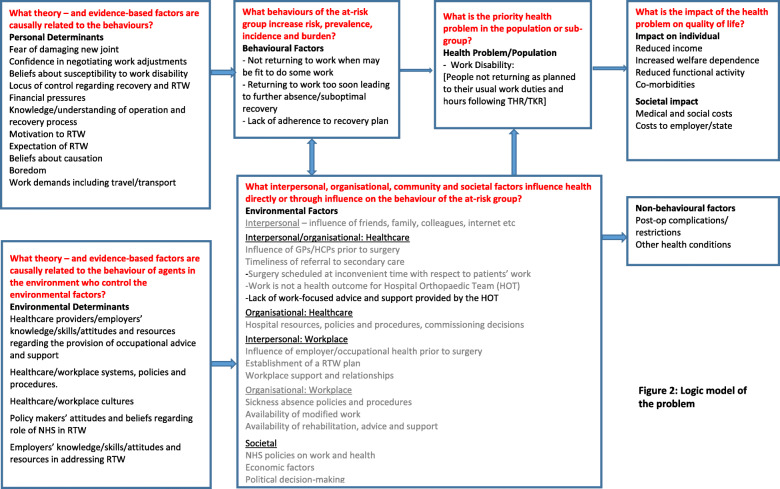
Logic model of the problem

These included interpersonal factors such as the influence of friends and family, interpersonal healthcare factors such as the influence and practice of primary care clinicians, organisational healthcare factors such as hospital resources, commissioning decisions, workplace factors such as the availability of modified work, and societal factors such as NHS policies regarding work and health outcome measurement. As the study had neither the remit nor resources to address all of the factors identified, the research team concluded that its main focus would be on the interpersonal (healthcare) factor of work-focused advice and support provided by hospital orthopaedic teams. The theory- and evidence- based factors causally related to the behaviour of hospital orthopaedic teams included their knowledge and skills in offering work-focused advice, attitudes and beliefs about roles and resources and patient need. The logic model (Fig. 2) illustrates in detail the problem under investigation and the relationships and factors associated with it.

It was agreed that the context of the intervention would be NHS Hospital Orthopaedic Teams consisting of surgeons, physiotherapists, occupational therapists, nurses and support staff. The goal was to design and develop an individualised occupational advice intervention that could be offered to any patient undergoing hip or knee replacement irrespective of their access to other occupational services.

### Step 2

In Step 2 the research team used the findings from Step 1 to specify who and/or what would need to change in order for patients to make a successful return to work following hip/knee replacement. The stated expected outcomes were agreed as follows:

1. The patient makes a safe and sustained return to usual work following surgery
2. The hospital orthopaedic team provides work-focused advice and support

The needs assessment described in Step 1, indicated that patients would benefit from occupational advice as early as possible in the hospital pathway, starting from the first clinic appointment with the surgeon. It should also involve employers and continue post-discharge. As well as containing generic information and advice, the intervention should also be individually targeted in order to reflect differing job demands and employment situations. A preliminary list of patient performance objectives and at what stage these might take place was drawn up by the research team (Additional file 4). Examples are shown in Table 2.

**Table 2:** Examples of preliminary patient performance objectives

Patient performance objective	Stage in pathway	Unresolved questions from Step 1
Patient is provided with advice and information about recovery and RTW	Following first clinic appointment/listing	What information is important? How and when will the information be delivered?
Patient identifies and prioritises potential barriers and solutions to a safe and appropriate RTW	Prior to surgery	How will patients do this? Will they do this with their employer? What skills will we need to equip them with?
Patient seeks help and support regarding RTW as required postoperatively	Following surgery	How do we facilitate this? What is the mechanism for support?

In order for patients to change their behaviour, and thus achieve their performance objectives, staff would also be required to change their behaviour. A preliminary list of staff performance objectives and at what stage these might take place were therefore also drawn up (Additional file 5). Examples are shown in Table 3.

**Table 3:** Examples of preliminary staff performance objectives

Staff performance objective	Stage in pathway	Unresolved questions from Step 1
Surgeon asks patients about their usual work and expectations of RTW following surgery	At first clinic appointment/listing	How do we ensure this is done? What tools can we develop to enable this process?
Staff provide ‘at risk’ patients with RTW checklist to complete with their employer	At listing	How do we identify ‘at risk’ patients and what tools could assist with this? What would the checklist include?
Staff summarise patient’s expected RTW outcome and RTW plan in ward discharge letter	Following surgery	How will junior doctors on the ward find this information? What specific information will be sent to the GP?

Drafting the performance objectives for patients and staff led to a number of unresolved questions (see right hand column of Tables 2 and 3). Uncertainty around these questions formed the basis of the initial draft questions put to a Delphi consensus group in Step 4.

Based on the literature, views and experiences of the research team, and the findings of the needs assessment, the key determinants (factors expected to influence behaviour) selected for both patients and hospital staff were:

- Knowledge & awareness
- Skills & self-efficacy
- Attitudes, beliefs, emotions
- Outcome expectations
- Perceived norms

The team specified the desired change objectives and built preliminary ‘matrices of change’ for every behaviour, target group and environmental agent that was required to be influenced. The preliminary performance objectives and matrices of change were revised and refined following the Delphi study (see Step 4). An example of the patient change objectives required to achieve a preliminary performance objective is shown in Table 4.

**Table 4:** Example of a patient change objective

Preliminary Performance Objective	Determinants				
	Knowledge & awareness	Skills & self-efficacy	Attitudes, beliefs, emotions	Outcome expectations	Perceived norms
Patient makes informed decision about surgery with respect to their work	Appraises the general risks/benefits of surgery and RTW rates. Appraises the likely impact of surgery on their ability to do their job. States that they have received sufficient information about surgery.	Expresses confidence in ability to make informed decision about surgery. Demonstrates ability to process information about surgical procedure and make informed choice.	Expresses willingness to take responsibility for surgical decision. Demonstrates appropriate response with regard to their decision.	Describes a realistic expectation of RTW outcome following surgery.	Perceives it is usual for patients to make an informed decision about surgery with respect to work. Recognises that nowadays patients are encouraged to take an active part in their care. Recognises that RTW is now considered a health outcome.

An example of the staff change objectives required to achieve a preliminary performance objective is shown in Table 5.

**Table 5:** Example of a staff change objective

Preliminary Performance Objective	Determinants				
	Knowledge & awareness	Skills & self-efficacy	Attitudes, beliefs, emotions	Outcome expectations	Perceived norms
Staff screen patients that intend to RTW to prior to meeting with surgeon using occupational checklist	Team members describe process of asking RTW patients to complete checklist and giving it to surgeon.	Team members express confidence in ability to ask RTW patients to complete checklist and giving it to surgeon	Team members state that asking RTW patients to complete occupational checklist will help patient and surgeon make more informed decision about surgery with regard to RTW	Team members recognise that preparing the patient and surgeon to discuss the patient’s RTW will aid their RTW	Team members perceived that preparing the patient and surgeon to discuss the patient’s RTW is usual practice

**Fig. 3:**
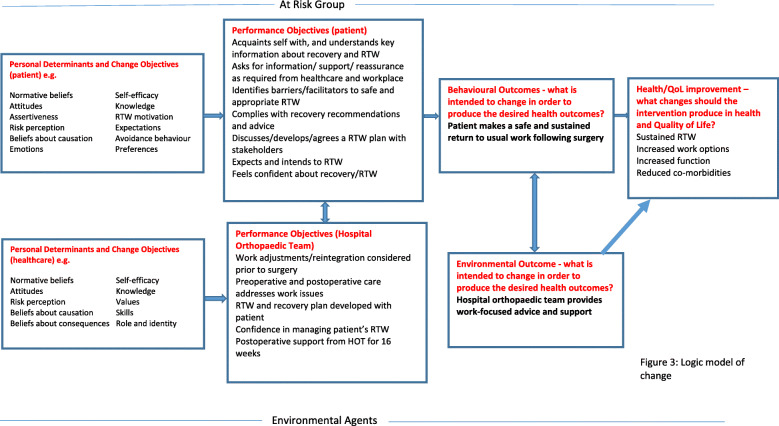
Logic model of change

A logic model of change was constructed to illustrate the proposed causal relations between theory- and evidence-based change methods, the determinants they were expected to influence, and behavioural and environmental outcomes that would address the problem (Fig. 3).

### Step 3

In this step the team consolidated their ideas about the components, scope and sequence of the intervention. Change objectives, organised by determinants in the matrices (factors expected to influence behaviour), were reviewed and theory- and evidence-based methods to influence the determinants in the desired direction were identified, following Intervention Mapping guidance [24, 35]. The parameters for each method were considered and the methods translated into practical applications that matched the target group (patients) (Additional file 6). An example is shown in Table 6.

**Table 6:** An example of parameters, methods of behaviour change, and practical applications for a patient determinant

Determinant: Knowledge and awareness				
**Change objective**	**Methods of behaviour change**	**Definition**	**Parameters**	**Application**
Patient identifies and prioritises potential barriers and solutions to a safe and appropriate RTW	Modelling *(Social Cognitive Theory)*	Providing an appropriate model	Identification with the model - receives positive reinforcement, coping vs. mastery model	Examples of other patients’ barriers and solutions and RTW plans included in workbook/on website and at preoperative presentations given by staff
	Variety of media/Elaboration *(Theory of Information Processing)*	Stimulating the learner to add meaning to the information that is processed	Messages that are personally relevant	Discussions with RTWC and preoperative education and assessment team

The same process was followed for Hospital Orthopaedic Team (HOT) staff (Additional file 7). An example is shown in Table 7.

**Table 7:** An example of parameters, methods of behaviour change, and practical applications for a staff determinant of behaviour

Determinant: Knowledge and awareness				
**Change objective**	**Methods of behaviour change**	**Definition**	**Parameters**	**Application**
Members of the outpatient clinic team know the process of identifying RTW patients before their appointment with surgeon: • how • when • where	Discussion *(Elaboration Likelihood Model)*	Encouraging consideration of topic in open formal debate.	Listening to learner to ensure correct schemas are activated.	Each member of team has own study pack containing this information.
	Providing Cues *(Theories of Information Processing)*	Assuring that the same cues are present at the time of learning and time of retrieval.	Work best when people select and provide own cues.	Study pack uses chunking, advance organisers and imagery methods to aid learning
	Individualisation /tailoring *(Trans-Theoretical Model)*	Matching to participant characteristics	Tailoring to participant, relevant to learner’s needs	Staff to suggest cues to action, e.g. posters/photos on ward/in clinic Tailored staff training

### Step 4

In Step 4 the team used a three-round modified Delphi process to address the areas of uncertainty around the preliminary patient and staff performance objectives and proposed intervention components.

In total 66 stakeholders including patients, employers, GPs, physiotherapists, occupational therapists and orthopaedic surgeons were invited to participate in the Delphi process. In Round 1 statements relating to the content of the intervention were explored. A total of 43 (65%) participants responded in Round 1, reaching consensus on 36 of the 64 statements presented. In Round 2 the intervention format, delivery, timing and measurement were examined with 26 (39%) participants responding. Consensus was reached for 49 of the 94 statements presented in Round 2. In Round 3 the finalised occupational advice intervention along with selected patient and staff materials were circulated and responses were received from 11 participants.

A detailed report of the Delphi process will be published separately but in summary the findings supported the OPAL intervention being embedded within usual care and with a multi-disciplinary team (MDT) approach. Roles and responsibilities for key staff groups already involved within the care pathway (Outpatient clinic staff, surgeons, ward nurses, ward doctors and therapy teams) were agreed. Additional roles were also created to deliver intervention components that the consensus group agreed were important but that could not be delivered by adapting the work of existing staff. This included the roles of a ‘return to work co-ordinator’ (RTWC) and deputy.

## Results

At the end of step 4 the occupational advice intervention and associated resources and training materials had been developed. The key elements of the final occupational advice intervention defined during this process were:

- Timing of the intervention including start and end points
- The patient identification process
- Delivery of information to the patient including its content, format, method / timing of delivery.
- Patient assessment and support by a designated member of the orthopaedic team
- Process of support, review and escalation based on patient need
- Mechanisms to support communication within the hospital team, between the hospital team and primary care, and between the patient and their employer:
- Training of the hospital orthopaedic team

To support the delivery of the intervention, a variety of resources were developed for patients and staff including: an interactive workbook for patients that included written information and also provided a mechanism by which they could develop an individualised return to work plan; written information for the employers, a telephone helpline for patients to call with issues relating to their return to work; a website containing return to work information; and examples of fit notes (a medical statement, issued in the UK, that provides evidence of the advice the patient has been given about their fitness to work), discharge letters (targeted at healthcare professionals) and RTW plans.

Central to the intervention was the development of the patient ‘return to work’ workbook. The workbook outlined a stepped process that allowed the patient to record individualised information about their own return to work process which they could then share with other members of the hospital orthopaedic team, their employer and their GP. The patient workbook and associated information for employers also described a variety of different mechanisms to facilitate safe and effective RTW. Mechanisms presented focussed on temporary workplace adaptions and phased returns and included information on: reduction in hours worked in the early phase of returning to work; altering patterns of work; adaption to the work environment; additional training to support new ways of working; improving accessibility and mobility; and colleague / line management support. Examples of how the developed materials promoted the desired change objectives, applications and overall message are given in Table 8.

**Table 8:** Examples of intervention resources

Proposed vehicle	Change objectives grouped by determinant	Methods and practical applications	Message content
Patient resource			
Return to Work workbook	*Knowledge and awareness*: knows key advice and information concerning recovery and RTW *Self-efficacy and skills*: able to acquaint self with key information about RTW *Attitudes,beliefs and expectations:* believes that revising RTW plan following surgery will aid RTW *Perceived norms*: recognises that RTW is now considered a positive health outcome	*Coherence and imagery*-sections of text have logical order and clearly related with graphics *Verbal persuasion* by Outpatient clinic staff and RTWC *Modelling* Provides examples of how patients have revised RTW plan *Consciousness raising:* information about causes/consequences	The HOT think that my RTW is important and that having this information will help The RTW book has been designed for and approved by patients as something they can use Other patients have revised their RTW plans and this has been helpful Working can have significant physical, mental and emotional health benefits, this is why the health service is focusing on it
Hospital Orthopaedic Team resource			
Examples of Return to Work Plans	*Knowledge and awareness*: Knowing what is expected from a completed template *Self-efficacy and skills*: Enabling the RTWC to support the patient	*Modelling*: appropriate examples provided for the RTWC to demonstrate completion *Facilitation:* creating an environment that makes the action easier	These are some typical examples based on real patient experiences These will help you support the patient plan their RTW

The OPAL team agreed upon a final version of thirteen patient performance objectives, nine prior to, and four post-surgery and twenty staff performance objectives, twelve prior to, and eight post-surgery (Additional files 8 and 9). These performance objectives underpinned the intervention describing what, when, how and why the specific elements would be delivered.

The final matrices of change and determinants for each patient and staff performance objective can be seen in Additional files 10 and 11.

## Discussion

To the authors knowledge this study is the first to report the methods used to develop an occupational advice intervention for delivery in the UK National Health Service to patients returning to work after hip and knee replacement surgery.

The intervention mapping approach proved complex and time-intensive, as has been reported elsewhere [36–38]. We also found it necessary to move backwards and forwards between some of the steps, for example the findings of the Delphi study in Step 4 helped us to review and finalise the initial performance objectives identified in Step 2. Bartholomew et al. [23] acknowledge that although IM is presented as a series of steps, it often needs to be an iterative process. However, the process did support the development of a clearly justified and structured intervention, and a strength of this study is that we were able to report each step of the process. It is unusual for IM studies focusing on return to work interventions to report on all six steps of the IM process, particularly Step 5. Fassier et al. [27] found that this step was insufficiently developed in any previous studies for it to be included in their review of fidelity to the IM protocol. Although we focus in this paper on steps 1–4 that describe the process for intervention development, steps 5 (implementation) and 6 (feasibility testing) have also been completed and these will be reported separately.

The intervention has a strong theoretical background and was underpinned by biopsychosocial models that supported behaviour change in the target groups (patients and stakeholders in the return to work process). It was manualised as a set of patient and staff performance objectives that defined its content, format, delivery and timing whilst maintaining pragmatism in the ability for participating sites to administer the intervention alongside standard care. Central to the intervention was the development of an interactive patient workbook that supported the self-directed development of a RTW plan, similar to other recently developed RTW interventions [39]. The intervention also shared many of the characteristics of the occupational advice interventions identified in our rapid evidence synthesis (step 1) including advice about job accommodation, mechanisms to support workplace visits and contact with the employer, education and advice, counselling and guidance through the RTWC, and involvement of the multidisciplinary team.

The study methodology employed during step 1allowed the OPAL investigators to collect a wide variety of data and perspectives across a number of NHS sites. It facilitated the collection of pertinent information about the target population and delivery of usual care, and explored outcomes of importance for this patient group.

### Applying the ecological model

In the OPAL study it became clear that the occupational advice intervention could only address outcomes based at the individual and interpersonal levels of the ecological model; it could not address outcomes based at organisational, community or societal levels. For example, it could not address NHS commissioning or primary care practice. It could not directly influence employer or workplace practice; however, it had the potential to indirectly make changes at these levels driven by changes in the individuals’ (employees’) behaviour. While the intervention focussed on the individual patient’s behaviour information was provided about workplace adaptions, phased returns and amended duties within both the patient and employer written materials. This information, combined with guidance within these written materials and from the RTWC role about the importance of involving employers in RTW planning meant that employers and, where present, their occupational health departments were indirectly integrated into the intervention. This was reinforced by the presence of the OPAL website containing all of the relevant RTW content from the developed resources which patients and their family, employers and their occupational health departments, GPs and hospital orthopaedic teams could access. This helped to broaden the scope of the intervention beyond the ‘individual’ and ensure these groups were not neglected. In their systematic review, Fassier et al. [27] concluded that IM is not a cast-iron solution to prevent theory and/or implementation failures of work disability prevention interventions. They have suggested that the limited number of effective interventions in work disability prevention indicate that IM needs to be adapted to reflect the complex interaction between healthcare and the workplace.

### Participatory planning group

Although the OPAL participatory planning group included an occupational physician and occupational psychologist, both with considerable appreciation and experience of workplace perspectives, the planning group did not include employers. As key stakeholders in RTW, it could be argued that employers should have been represented more directly in the group, however as discussed above, in this study it would not have been possible to impact on the individual patient’s employer behaviours, other than indirectly through the patient themselves. The employer perspective was however sought/represented in steps 1 (needs assessment) and step 4 (Delphi study).

The process of designing the OPAL occupational advice intervention using intervention mapping, prior to implementation in the feasibility study, took 24 months. At times the volume of information generated was overwhelming, and having three different teams based at different locations added to the complexities of project management. These experiences underlined the importance of having sufficient resources and frequent planning meetings in conducting the IM process.

### Comparison with other studies

There are other established methods that could be applied to intervention development in RTW, such as the PRECEDE-PROCEED model [40]. Two other frameworks have been developed with the purpose of linking theory to behaviour change. One is the Behaviour Change Wheel (BCW) [41], a synthesis of 19 theoretical frameworks of behaviour change, the other is the Theoretical Change Framework [42] consisting of 14 domains of theoretical constructs. These have recently been used in the field of occupational health to better understand occupational physicians’ behaviours regarding temporary work modifications in RTW [43], however to our knowledge these have not been used to develop RTW interventions.

As in the intervention developed by Noordegraaf et al. [30], the OPAL intervention began preoperatively in order to utilise the period between listing and surgery to start planning the RTW process. However, while Nooredegraaf et al. made a pre-mapping decision to develop an eHealth intervention, the findings of the Step 1 needs assessment in OPAL indicated a more individually-targeted approach with paper-based materials was required.

The majority of other RTW interventions using IM have identified performance objectives (POs) and change objectives (COs) only at the level of the worker or patient. Of the six studies promoting RTW reviewed by Fassier et al. [27], only those by Amendolia et al. [37] and Desiron et al. [44] identified POs and COs for other RTW stakeholders. Ammendolia et al. identified POs and COs for workplace-based stakeholders, including supervisors and a RTWC, to support those with low back pain RTW. Desiron et al. identified POs and COs for Occupational Therapists delivering an intervention for breast cancer patients. Although Noordegraaf et al. identified POs for stakeholders, they did not report what changes would be required in order for these POs to be realised. In OPAL, in order to affect patient change, and a necessary change of culture within the hospital orthopaedic team, it was clear that performance and change objectives were also required for those delivering the intervention. This included surgeons, outpatient clinic staff, therapy and nursing staff, and the Return to Work Co-ordinator.

### Generalisability of the intervention to other healthcare settings

The OPAL team was commissioned to develop an intervention for patients within the UK National Health Service (NHS) system. As a result, the needs assessment described in step 1 collected data from this healthcare setting through the stakeholder interviews, cohort study and survey of practice. This information, alongside the evidence synthesis, formed the basis for the logic model and subsequent intervention development. The UK has nationalised healthcare and welfare systems and this setting will have directly impacted on development of this intervention which was specifically designed for delivery within the NHS. This may limit the transferability of the intervention to other healthcare systems that have different models for funding and delivery [45]. However, we believe that while some performance objectives that define the intervention may not be generalisable to other healthcare settings, many of the intervention’s core principles are transferable. These include the principles of: early patient identification; delivery of key information to patients and their employers; assessment and support by a member of the orthopaedic team; procedures for escalation based on patient need; mechanisms to support communication; and training and support for the clinical teams delivering care.

## Conclusions

Following the IM protocol resulted in a structured and justified occupational intervention for delivery in secondary care for patients undergoing total hip and knee replacement. Results from the feasibility testing may provide further information about the effectiveness and cost-effectiveness of the intervention.

## Supplementary information

**Additional file 1.** Example cohort questionnaire: Baseline hip questionnaire.

**Additional file 2.** Example interview guide: Patient interview guide.

**Additional file 3.** Summary of the key information developed from IM Step 1, based on the PICO format.

**Additional file 4.** Preliminary list of patient performance objectives.

**Additional file 5.** Preliminary list of staff performance objectives.

**Additional file 6.** Parameters, methods and practical applications for patient determinants

**Additional file 7.** Parameters, methods and practical applications for staff determinants

**Additional file 8.** Final patient performance objectives

**Additional file 9.** Final performance objectives for members of the Hospital Orthopaedic Team

**Additional file 10.** Final matrices of change and determinants for each patient performance objective

**Additional file 11.** Final matrices of change and determinants for each Hospital Orthopaedic Team performance objective

## Data Availability

The datasets used and/or analysed during the current study are available from the corresponding author on reasonable request.

## Abbreviations

CO: Change objective
GP: General practitioner
HOT: Hospital orthopaedic team
IM: Intervention mapping
MDT: Multi-disciplinary team
NHS: National health service
OPAL: Occupational advice for Patients undergoing arthroplasty of the lower limb
PI: Principal investigator
PICO: Population, intervention, comparator, outcome
PO: Performance objective
RTW: Return to work
RTWC: Return to work coordinator
THR: Total hip replacement
TKR: Total knee replacement
UK: United Kingdom
