# Supplementary material for: Using intervention mapping to develop an occupational advice intervention to aid return to work following hip and knee replacement in the United Kingdom
Source: BMC Health Serv Res. 2020 Jun 9;20:523. doi: 10.1186/s12913-020-05375-3 (PMC7285551; doi:10.1186/s12913-020-05375-3)
Supplement: Supplementary file 1 — Additional file 1. Example cohort questionnaire: Baseline hip questionnaire. [file 12913_2020_5375_MOESM1_ESM.docx]

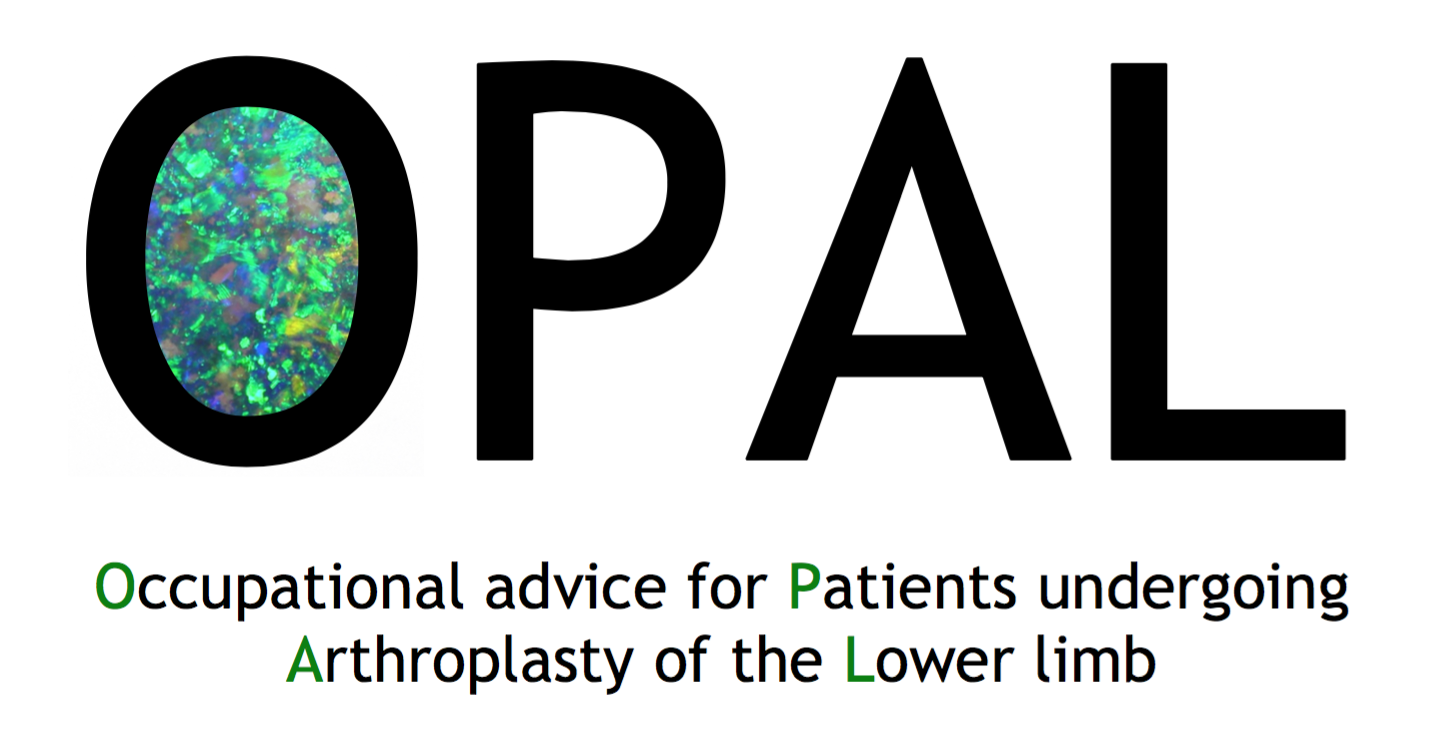


**BASELINE HIP QUESTIONNAIRE**

**FOR STUDY PARTICIPANT COMPLETION**

**TO BE COMPLETED BY RESEARCH TEAM**

| Site identifier: |  |
| --- | --- |
| Study ID number: |  |
| Date of questionnaire completion: | ⬜ ⬜ / ⬜ ⬜ / ⬜ ⬜  (Day / Month / Year) |
| Date of surgery: | ⬜ ⬜ / ⬜ ⬜ / ⬜ ⬜  (Day / Month / Year) |

**Section 1: Patient details**

**The following questions ask general information about you:**

1. Age ⬜ ⬜ ⬜ years

2. Gender: Male ⬜ Female ⬜

3. Height ⬜ ⬜ ⬜ cm or ⬜ feet, ⬜ ⬜ inches

4. Weight ⬜ ⬜ ⬜ kg or ⬜ ⬜ stones, ⬜ ⬜ pounds

5. Postcode (First part only e.g. TS16, NR6, NG24) ⬜ ⬜ ⬜ ⬜

6. How would you describe your ethnic status?

(Please Tick one box only)

White (e.g. British or Irish) ⬜

Asian (e.g. Indian, Pakistani, Bangladeshi) ⬜

Black (e.g. African or Caribbean) ⬜

Oriental (e.g. Chinese, Japanese or Korean) ⬜

Other (Please state)

7. How would you best describe your current living arrangements and family support?

(Please Tick one box only)

Living with spouse or partner ⬜

Living with family ⬜

Living with friends ⬜

Living alone ⬜

Other (Please state)

8. Which of the following statement best describes the home support available to you following your operation (This may include relatives and friends living nearby and providing support)?

(Please Tick one box only)

Support from spouse or partner ⬜

Support from family ⬜

Support from friends ⬜

I have no direct support from friends and family ⬜

I am a carer for other family members ⬜

Other (Please state)

**Section 2: Physical, Mental Health and Wellbeing**

**The following questions ask about your health and wellbeing:**

| 1. Do you consider yourself to have a chronic medical condition (e.g. Heart disease, Diabetes, Airways disease, Kidney disease or Liver disease)? | Yes ⬜ No ⬜ |
| --- | --- |
| 2. Do you have problems with your other hip joint? | Yes ⬜ No ⬜ |
| 3. Do you have problems with your knee joints? | Yes ⬜ No ⬜ |
| 4. Do you suffer from chronic back or neck problems | Yes ⬜ No ⬜ |

5. Over the **past 2 weeks**, how often have you been bothered by any of the following problems? (Please Tick one box per row)

|  | Not at all | Several days | More than half the days | Nearly every day |
| --- | --- | --- | --- | --- |
| - Little interest or pleasure doing things | ⬜ | ⬜ | ⬜ | ⬜ |
| - Feeling down, depressed or hopeless | ⬜ | ⬜ | ⬜ | ⬜ |
| - Trouble falling asleep, staying asleep, or sleeping too much | ⬜ | ⬜ | ⬜ | ⬜ |
| - Poor appetite or overeating | ⬜ | ⬜ | ⬜ | ⬜ |
| - Feeling tired or having little energy | ⬜ | ⬜ | ⬜ | ⬜ |
| - Feeling bad about yourself – or that you’re a failure or have let yourself or your family down | ⬜ | ⬜ | ⬜ | ⬜ |
| - Trouble concentrating on things, such as reading the newspaper or watching television | ⬜ | ⬜ | ⬜ | ⬜ |
| - Moving or speaking so slowly that other people could have noticed. Or, the opposite – being so fidgety or restless that you have been moving around a lot more than usual | ⬜ | ⬜ | ⬜ | ⬜ |
| - Thoughts that you would be better off dead or of hurting yourself in some way | ⬜ | ⬜ | ⬜ | ⬜ |
| - Feeling nervous, anxious or on edge | ⬜ | ⬜ | ⬜ | ⬜ |
| - Not being able to stop or control worrying | ⬜ | ⬜ | ⬜ | ⬜ |

**If you ticked any problems, how difficult have those problems made it for you to do your work, take care of things at home, or get along with other people?**

Not difficult at all ⬜ Somewhat difficult ⬜ Very difficult ⬜ Extremely difficult ⬜

**The following questions are about how you deal with stress and change:**

6. Please respond to the following 6 statements by marking one box per row:

| Statement | Strongly disagree | Disagree | Neutral | Agree | Strongly Agree |
| --- | --- | --- | --- | --- | --- |
| I tend to bounce back quickly after hard times | ⬜ | ⬜ | ⬜ | ⬜ | ⬜ |
| I have a hard time making it through stressful events | ⬜ | ⬜ | ⬜ | ⬜ | ⬜ |
| It does not take me long to recover from a stressful event | ⬜ | ⬜ | ⬜ | ⬜ | ⬜ |
| It is hard for me to snap back when something bad happens | ⬜ | ⬜ | ⬜ | ⬜ | ⬜ |
| I usually come through difficult times with little trouble | ⬜ | ⬜ | ⬜ | ⬜ | ⬜ |
| I tend to take a long time to get over set-backs in my life | ⬜ | ⬜ | ⬜ | ⬜ | ⬜ |

**Section 3: Employment details**

**The following questions ask you about your usual job:**

1. In your own words please describe your job title / the work you do or have done within the last six months?

2. Which of these best describes your usual work?

(Please Tick one box only)

Employed Full-time ⬜

Employed Part-time ⬜

Self employed ⬜

Unpaid work – Carer, volunteer, housework ⬜

Other ⬜

3. Please record the number of hours you work each week (when doing your usual job(s))?

(Please record number of hours to the nearest hour)

Employed Full-time ⬜ ⬜ hours

Employed Part-time ⬜ ⬜ hours

Self employed ⬜ ⬜ hours

Unpaid work – Carer, volunteer, housework ⬜ ⬜ hours

Other ⬜ ⬜ hours

4. Which of the descriptions below best describes your employer?

(Please Tick one box only)

Large employer (employs more than 250 people) ⬜

Medium sized employer (employs between 50 and 250 people) ⬜

Small employer (employs between 10 and 49 people) ⬜

Micro employer (employs between 2 and 9 people) ⬜

I work alone ⬜

5. Is your employer?

(Please Tick one box only)

A public sector employer ⬜

A private sector employer ⬜

Don’t know / Unsure ⬜

Other (Please state)

6. How long have you been in your current job? ⬜ ⬜ years & ⬜ ⬜ months

7. As part of your job are you required to work rotating shifts? Yes ⬜ No ⬜

8. Do you drive yourself to work? Yes ⬜ No ⬜

9. Do you have to drive while at work? Yes ⬜ No ⬜

**10. Please answer the following questions about your usual job:**

The questions below concern characteristics of your job. Using the scale below, please indicate the extent to which you agree with each statement. Remember to think only about your job itself, rather than your reactions to the job (Please Tick one box per row).

|  | Strongly Disagree | Disagree | Neither Agree nor Disagree | Agree | Strongly  Agree |
| --- | --- | --- | --- | --- | --- |
| 1. The job allows me to make my own decisions about how to schedule my work. | ⬜ | ⬜ | ⬜ | ⬜ | ⬜ |
| 2. The job allows me to decide on the order in which things are done on the job. | ⬜ | ⬜ | ⬜ | ⬜ | ⬜ |
| 3. The job allows me to plan how I do my work. | ⬜ | ⬜ | ⬜ | ⬜ | ⬜ |
| 1. The seating arrangements on the job are adequate (e.g., ample opportunities to sit, comfortable chairs, good postural support). | ⬜ | ⬜ | ⬜ | ⬜ | ⬜ |
| 2. The work place allows for all size differences between people in terms of clearance, reach, eye height, leg room, etc. | ⬜ | ⬜ | ⬜ | ⬜ | ⬜ |
| 3. The job involves excessive reaching. | ⬜ | ⬜ | ⬜ | ⬜ | ⬜ |
| 1. The job requires a great deal of muscular endurance. | ⬜ | ⬜ | ⬜ | ⬜ | ⬜ |
| 2. The job requires a great deal of muscular strength. | ⬜ | ⬜ | ⬜ | ⬜ | ⬜ |
| 3. The job requires a lot of physical effort. | ⬜ | ⬜ | ⬜ | ⬜ | ⬜ |
| 1. I have the opportunity to develop close friendships in my job. | ⬜ | ⬜ | ⬜ | ⬜ | ⬜ |
| 2. I have the chance in my job to get to know other people. | ⬜ | ⬜ | ⬜ | ⬜ | ⬜ |
| 3. I have the opportunity to meet with others in my work. | ⬜ | ⬜ | ⬜ | ⬜ | ⬜ |
| 4. My supervisor is concerned about the welfare of the people that work for him/her. | ⬜ | ⬜ | ⬜ | ⬜ | ⬜ |
| 5. People I work with take a personal interest in me. | ⬜ | ⬜ | ⬜ | ⬜ | ⬜ |
| 6. People I work with are friendly. | ⬜ | ⬜ | ⬜ | ⬜ | ⬜ |

**Section 4: Working before your operation**

**The following questions ask you about your ability to work (including unpaid work such as volunteering or acting as a carer) in the last 6 months and any changes to your working pattern in the months before your surgery:**

| 1. When was the last day you worked prior to your surgery? (Day / Month / Year) | ⬜ ⬜ / ⬜ ⬜ / ⬜ ⬜ |
| --- | --- |
| 2. Where you working in your usual role (normal hours and duties) right up to your last day at work before your operation?  **If No,** | Yes ⬜ No ⬜ |
| - Please select which of the following options best describes how you have been working prior to your surgery. (Please Tick one box only) | Reduced hours, usual duties ⬜  Usual hours, amended duties ⬜  Reduced hours and amended duties ⬜ |
| - If you were working reduced hours before you left work:  1. How many hours per week were you working?   (Please record number of hours to the nearest hour)   1. For how many weeks had you been working reduced hours?  - If you were working on amended duties before you left work:  1. For how many weeks had you been working on amended duties? | ⬜ ⬜ hours  ⬜ ⬜ weeks  ⬜ ⬜ weeks |
| 3. Have you had any periods of sick leave in the 6 months prior to your operation?  **If Yes,** | Yes ⬜ No ⬜ |
| - How many separate periods of sick leave have you had because of the joint that requires surgery? | ⬜ ⬜ sick leave periods |
| - How many separate periods of sick leave have you had for other reasons? | ⬜ ⬜ sick leave periods |
| - Approximately how many days work have you missed in the last 6 months because of the joint that requires replacement surgery? | ⬜ ⬜ ⬜ days |
| - Approximately how many days work have you missed in the last 6 months because of other reasons? | ⬜ ⬜ ⬜ days |
| 4. Is there a sickness absence policy in your place of work? | Yes ⬜ No ⬜  Don’t know / Unsure ⬜ |
| 5. Do you receive any of the following payments during periods of sick leave? (Please tick all that apply) | Statutory sick pay ⬜  Employer based sick pay ⬜  Don’t know / Unsure ⬜ |
| - If you do receive sickness payments, for how long do you receive them? | < 1 month ⬜  1-3 months ⬜  3-6 months ⬜  >6 months ⬜  Don’t know / Unsure ⬜ |
| 6. Were any changes (adaptations) made to your workplace to allow you to do your job in the 6 months before your operation?  **If Yes,** **please give details below** | Yes ⬜ No ⬜ |

**Section 5: The advice and care you received before your operation**

**The following questions ask about your access to advice before your operation**

| 1. Do you have access to an occupational health service through your employer? | Yes ⬜ No ⬜  Don’t know / Unsure ⬜ |
| --- | --- |
| 2. Have you received any advice from any individual or organisation about returning to work following your operation? | Yes ⬜ No ⬜  Don’t know / Unsure ⬜ |

🡪 If you received advice about returning to work, whom did you receive it from?

Surgeon ⬜

G.P ⬜

Occupational health ⬜

Physiotherapist ⬜

Occupational therapist ⬜

Employer (e.g. supervisor, manager, human resources) ⬜

Other (Please state)

| 3. Have you received any advice about when it is safe to start driving after your operation? | Yes ⬜ No ⬜  Don’t know / Unsure ⬜ |
| --- | --- |

**The following questions ask about your expectation of returning to work and usual activities after surgery**

4. How long do you think it will be before **you are ready** to return to **work** after your operation?

⬜ ⬜ weeks

5. How long do you think it will be before **your employer** is happy for you to return to **work** after your operation?

⬜ ⬜ weeks

6. How long do you think it will be before **you are ready** to return to your **usual daily activities** after your operation?

⬜ ⬜ weeks

7. How long do you think it will be before **you are ready** to **drive** after your operation?

⬜ ⬜ weeks

**Section 6: Health care use**

**The following questions ask about the health care you have received over the PAST EIGHT WEEKS. They ask about the health care you have received for your hip and the health care you have received for other reasons. Please record the number of times you have come in to contact with each of the health care teams listed in the boxes below.**

Over **the past eight weeks**, how many times have you:

| **NHS OUT OF HOSPITAL CARE** | **About your joint replacement**  (If none enter ‘0’) | **For another reason**  (If none enter ‘0’) |
| --- | --- | --- |
| 1. Seen a GP at your GP practice? | ⬜⬜ | ⬜⬜ |
| 1. Been seen by a GP at home? | ⬜⬜ | ⬜⬜ |
| 1. Seen a nurse at your GP practice? | ⬜⬜ | ⬜⬜ |
| 1. Been seen by a community nurse at home? | ⬜⬜ | ⬜⬜ |
| 1. Seen an occupational therapist? | ⬜⬜ | ⬜⬜ |
| 1. Seen a physiotherapist? | ⬜⬜ | ⬜⬜ |
| 1. Had an appointment with any other health service professional? | ⬜⬜ | ⬜⬜ |
| **CARE FROM THE NHS IN HOSPITAL** | **About your joint replacement**  (If none enter ‘0’) | **For another reason**  (If none enter ‘0’) |
| 1. How many nights have you stayed in hospital as an **in-patient**? ***(admitted and discharged on a different day)*** | ⬜⬜ | ⬜⬜ |
| 1. Visited hospital as a **day case?** ***(admitted and discharged in the same day)***  *e.g. admitted at 2am and discharged at 10am OR admitted at 8am and discharged at 10pm* | ⬜⬜ | ⬜⬜ |
| 1. Attended a **hospital clinic** as an outpatient? | ⬜⬜ | ⬜⬜ |
| 1. Visited **Accident and Emergency**? | ⬜⬜ | ⬜⬜ |
| 1. Attended **physiotherapy** at hospital? | ⬜⬜ | ⬜⬜ |

**Section 7: Health questionnaires**

**These questions ask about the impact your painful joint has on your daily activities and quality of life**

Please answer the following 12 questions about your hip. Choose only one answer per question. Please only consider how you have been getting on during the **past four weeks**

| **How would you describe the pain you usually have in your hip?** | **Score** | **Have you been able to put on a pair of socks, stockings or tights?** | **Score** |
| --- | --- | --- | --- |
| None – **4** |  | Yes, easily – **4** |  |
| Very mild – **3** |  | With little difficulty – **3** |  |
| Mild – **2** |  | With moderate difficulty – **2** |  |
| Mild moderate – **1** |  | With extreme difficulty – **1** |  |
| Severe – **0** |  | No, impossible – **0** |  |
|  |  |  |  |
| **Have you been troubled by pain from your hip in bed at night?** |  | **After a meal (sat at a table), how painful has it been for you to stand up from a chair because of your hip?** |  |
| No nights – **4** |  | Not at all painful – **4** |  |
| Only 1 or 2 nights – **3** |  | Slightly painful – **3** |  |
| Some nights – **2** |  | Moderately painful – **2** |  |
| Most nights – **1** |  | Very painful – **1** |  |
| Every night – **0** |  | Unbearable – **0** |  |
|  |  |  |  |
| **Have you had any sudden, severe pain-' shooting ', 'stabbing', or 'spasms' from your affected hip?** |  | **Have you had any trouble getting in and out of a car or using public transportation because of your hip?** |  |
| No days – **4** |  | No trouble at all – **4** |  |
| 1 or 2 days – **3** |  | Very little trouble – **3** |  |
| Some days – **2** |  | Moderate trouble – **2** |  |
| Most days – **1** |  | Extreme difficulty – **1** |  |
| Every day – **0** |  | Impossible to do – **0** |  |
|  |  |  |  |
| **Have you been limping when walking because of your hip?** |  | **Have you had any trouble with washing and drying yourself (all over) because of your hip?** |  |
| Rarely / Never – **4** |  | No trouble at all – **4** |  |
| Sometimes or just at first – **3** |  | Very little trouble – **3** |  |
| Often, not just at first – **2** |  | Moderate trouble – **2** |  |
| Most of the time – **1** |  | Extreme difficulty – **1** |  |
| All of the time – **0** |  | Impossible to do – **0** |  |
|  |  |  |  |
| **For how long have you been able to walk before the pain in your hip becomes severe (with or without a walking aid)?** |  | **Could you do the household shopping on your own?** |  |
| No pain, even after more than 30 minutes – **4** |  | Yes, easily – **4** |  |
| 16-30 minutes – **3** |  | With little difficulty – **3** |  |
| 5-15 minutes – **2** |  | With moderate difficulty – **2** |  |
| Around the house only – **1** |  | With extreme difficulty – **1** |  |
| Unable to walk at all – **0** |  | No, impossible – **0** |  |
|  |  |  |  |
| **Have you been able to climb a flight of stairs?** |  | **How much has pain from your hip interfered with your usual work, including housework?** |  |
| Yes, easily – **4** |  | Not at all – **4** |  |
| With little difficulty – **3** |  | A little bit – **3** |  |
| With moderate difficulty – **2** |  | Moderately – **2** |  |
| With extreme difficulty – **1** |  | Greatly – **1** |  |
| No, impossible – **0** |  | Totally – **0** |  |

**The next pages ask the same questions twice; once about your health today and once about your health at 4 weeks before your hip replacement operation.**

**YOUR HEALTH TODAY:**

Under each heading, please tick the **ONE** box that best describes your health **TODAY**

**MOBILITY**

I have no problems in walking about ⬜

I have slight problems in walking about ⬜

I have moderate problems in walking about ⬜

I have severe problems in walking about ⬜

I am unable to walk about ⬜

**SELF-CARE**

I have no problems washing or dressing myself ⬜

I have slight problems washing or dressing myself ⬜

I have moderate problems washing or dressing myself ⬜

I have severe problems washing or dressing myself ⬜

I am unable to wash or dress myself ⬜

**USUAL ACTIVITIES** *(e.g. work, study, housework, family or leisure activities)*

I have no problems doing my usual activities ⬜

I have slight problems doing my usual activities ⬜

I have moderate problems doing my usual activities ⬜

I have severe problems doing my usual activities ⬜

I am unable to do my usual activities ⬜

**PAIN / DISCOMFORT**

I have no pain or discomfort ⬜

I have slight pain or discomfort ⬜

I have moderate pain or discomfort ⬜

I have severe pain or discomfort ⬜

I have extreme pain or discomfort ⬜

**ANXIETY / DEPRESSION**

I am not anxious or depressed ⬜

I am slightly anxious or depressed ⬜

I am moderately anxious or depressed ⬜

I am severely anxious or depressed ⬜

I am extremely anxious or depressed ⬜

*UK (English) v.2 © 2009 EuroQol Group. EQ-5D™ is a trade mark of the EuroQol Group*

The best health you can imagine

10

0

20

30

40

50

60

80

70

90

100

5

15

25

35

45

55

75

65

85

95

| We would like to know how good or bad your health is TODAY. |
| --- |
| This scale is numbered from 0 to 100. |
| 100 means the best health you can imagine. 0 means the worst health you can imagine. |
| Mark an X on the scale to indicate how your health is TODAY. |
| Now, please write the number you marked on the scale in the box below. |

The worst health you can imagine

*UK (English) © 2009 EuroQol Group EQ-5D™ is a trade mark of the EuroQol Group*

YOUR HEALTH TODAY =

Please think back to your health **before** your hip replacement operation.

Under each heading, please tick the **ONE** box that best describes your health **4 WEEKS BEFORE YOUR OPERATION**

**MOBILITY**

I have no problems in walking about ⬜

I have slight problems in walking about ⬜

I have moderate problems in walking about ⬜

I have severe problems in walking about ⬜

I am unable to walk about ⬜

**SELF-CARE**

I have no problems washing or dressing myself ⬜

I have slight problems washing or dressing myself ⬜

I have moderate problems washing or dressing myself ⬜

I have severe problems washing or dressing myself ⬜

I am unable to wash or dress myself ⬜

**USUAL ACTIVITIES** *(e.g. work, study, housework, family or leisure activities)*

I have no problems doing my usual activities ⬜

I have slight problems doing my usual activities ⬜

I have moderate problems doing my usual activities ⬜

I have severe problems doing my usual activities ⬜

I am unable to do my usual activities ⬜

**PAIN / DISCOMFORT**

I have no pain or discomfort ⬜

I have slight pain or discomfort ⬜

I have moderate pain or discomfort ⬜

I have severe pain or discomfort ⬜

I have extreme pain or discomfort ⬜

**ANXIETY / DEPRESSION**

I am not anxious or depressed ⬜

I am slightly anxious or depressed ⬜

I am moderately anxious or depressed ⬜

I am severely anxious or depressed ⬜

I am extremely anxious or depressed ⬜

*UK (English) v.2 © 2009 EuroQol Group. EQ-5D™ is a trade mark of the EuroQol Group*

The best health you can imagine

10

0

20

30

40

50

60

80

70

90

100

5

15

25

35

45

55

75

65

85

95

| We would like to know how good or bad your health was 4 WEEKS BERORE YOUR OPERATION. |
| --- |
| This scale is numbered from 0 to 100. |
| 100 means the best health you can imagine. 0 means the worst health you can imagine. |
| Mark an X on the scale to indicate how your health was 4 WEEKS BEFORE YOUR OPERATION. |
| Now, please write the number you marked on the scale in the box below. |

YOUR HEALTH 4 WEEKS BEFORE YOUR OPERATION =

The worst health you can imagine

*UK (English) © 2009 EuroQol Group EQ-5D™ is a trade mark of the EuroQol Group*


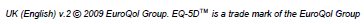


**Section 8: Workplace questionnaires**

**The following questions ask about how your joint problems interfered with your ability to do your job in the weeks before your operation.**

Health problems can make it difficult for working people to perform certain parts of their jobs. We are interested in learning about how your health may have affected you at work **during the 2 weeks before your operation (IF YOU WERE NOT WORKING IN THE LAST 2 WEEKS PLEASE LEAVE THIS SECTION BLANK).** These questions will ask you to think about your physical health and emotional problems. These refer to any ongoing permanent medical conditions you may have and the effects of any treatments you are taking for these. Emotional problems may include feeling depressed or anxious.

1. **Time management: In the 2 weeks before your operation, how much of the time did your physical health or emotional problems make it difficult for you to do the following?**

**Get going easily at the beginning of the day:**

Difficult all of the time (100%) ⬜

Difficult most of the time ⬜

Difficult some of the time (about 50%) ⬜

Difficult a slight bit of the time ⬜

Difficult none of the time (0%) ⬜

Does not apply to my job ⬜

**Start your job on time as soon as you arrived at work**

Difficult all of the time (100%) ⬜

Difficult most of the time ⬜

Difficult some of the time (about 50%) ⬜

Difficult a slight bit of the time ⬜

Difficult none of the time (0%) ⬜

Does not apply to my job ⬜

1. **Physical tasks:**

**In the 2 weeks before your operation, how much of the time were you able to sit, stand, or stay in once position for longer than 15 minutes while working, without difficulty caused by physical health or emotional problems?**

Able all of the time (100%) ⬜

Able most of the time ⬜

Able some of the time (about 50%) ⬜

Able a slight bit of the time ⬜

Able none of the time (0%) ⬜

Does not apply to my job ⬜

**In the 2 weeks before your operation, how much of the time were you able to repeat the same motions over and over again while working, without difficulty caused by physical health or emotional problems?**

Able all of the time (100%) ⬜

Able most of the time ⬜

Able some of the time (about 50%) ⬜

Able a slight bit of the time ⬜

Able none of the time (0%) ⬜

Does not apply to my job ⬜

1. **Concentration and interpersonal relationships:**

**In the 2 weeks before your operation, how much of the time did your physical health or emotional problems make it difficult for you to concentrate on your work?**

Difficult all of the time (100%) ⬜

Difficult most of the time ⬜

Difficult some of the time (about 50%) ⬜

Difficult a slight bit of the time ⬜

Difficult none of the time (0%) ⬜

Does not apply to my job ⬜

**In the 2 weeks before your operation, how much of the time did your physical health or emotional problems make it difficult for you to speak with people in-person, in meetings or on the phone?**

Difficult all of the time (100%) ⬜

Difficult most of the time ⬜

Difficult some of the time (about 50%) ⬜

Difficult a slight bit of the time ⬜

Difficult none of the time (0%) ⬜

Does not apply to my job ⬜

1. **Work output: In the 2 weeks before your operation, how much of the time did your physical health or emotional problems make it difficult for you to do the following?**

**Handle your workload:**

Difficult all of the time (100%) ⬜

Difficult most of the time ⬜

Difficult some of the time (about 50%) ⬜

Difficult a slight bit of the time ⬜

Difficult none of the time (0%) ⬜

Does not apply to my job ⬜

**Finish work on time:**

Difficult all of the time (100%) ⬜

Difficult most of the time ⬜

Difficult some of the time (about 50%) ⬜

Difficult a slight bit of the time ⬜

Difficult none of the time (0%) ⬜

Does not apply to my job ⬜

**Thank you for completing this questionnaire.**

**We would be grateful if you could spend a few minutes checking your answers and that you have responded to every question.**

**PLEASE RETURN YOUR QUESTIONNAIRE TO A MEMBER OF THE RESEARCH TEAM BEFORE DISCHARGE.**
