# Supplementary material for: Using intervention mapping to develop an occupational advice intervention to aid return to work following hip and knee replacement in the United Kingdom
Source: BMC Health Serv Res. 2020 Jun 9;20:523. doi: 10.1186/s12913-020-05375-3 (PMC7285551; doi:10.1186/s12913-020-05375-3)
Supplement: Supplementary file 2 — Additional file 2. Example interview guide: Patient interview guide. [file 12913_2020_5375_MOESM2_ESM.docx]

**Patient interview guide**

- Thank you for taking the time to meet with us today.
- Have you read the information leaflet and informed consent form? Do you have any questions before we start? There are no right or wrong answers and you don’t have to answer any questions that you don’t feel comfortable talking about. If it’s ok with you, we will use a digital audio recorder to ensure that the interview is accurately documented. Everything you say will be kept confidential and anonymous. Your name will not be mentioned on any published documents, and therefore anything you say cannot be identified as coming from you. Any names of individuals or places that you might refer to during the interview will be anonymised when transcribed. The recording will be stored securely at the University of Nottingham.
- You are welcome to request a copy of your interview transcript if you would like to review it for clarification, to add to it, or to indicate that all or part of it should not be used.

| **Topic area** | **Question** | **Prompts** |
| --- | --- | --- |
|  | | |
| Personal information | Can you tell me about yourself? | Health, home, family, work, hobbies, interests. Usual work and other activities. Driving (may be relevant to work) |
| Relevant experience | What has been your experience of RTW/RUA following knee or hip replacement? | Who else has been involved –e.g. GP, AHPs, OH, managers, HR, Fit for Work Services, family  When and how are they involved – how effective are they?  What has happened re RTW/RUA and when? |
| Perceived obstacles/facilitators | What things make/could make it difficult for patients who have had knee or hip replacement to RTW/RUA?  What things make/could make it easier for patients who have had knee or hip replacement to RTW/RTUA?  What helps/would help you and other patients to RTW/RUA? | Information-related  Extent of of sufficient/consistent/useful information/advice for patients/GPs/employers on RTW/RUA following surgery. What needed? How information/advice should be delivered/accessed? When? By whom?  Patient-related  Motivation/attitude/beliefs/expectations re RTW/RUA including self-efficacy, anxiety.  Sick leave history/absence  Extent of functional ability prior to surgery  Life context – age, home circumstances, travel to work. Family roles and responsibilities. Work-life balance. Financial circumstances.  Transferable skills  Co-morbidities  Adherence/compliance with advice/support  Symptom management  Workplace-related  Relationships  Workplace/managerial support  Workplace conditions – environment, pace, tasks, job demands, shifts/hours, travel, availability of adjustments/redeployment, culture, sick pay/absence policies.  Equipment  Activity outside workplace  Physical and mental demands of UA (including PADL, DADL, hobbies, interests, family roles and responsibilities, social activities)  RTW management  Communication/transfer of information between key players. How conducted, by who, when?  Extent of an agreed RTW plan  Co-ordination of RTW  Key players’ skill in RTW management  Societal  Economic factors/conditions – local, national  Surgery related  Waiting times/delays to surgery  Surgical approach/type of operation/components used  Experience/ability of surgeon  Complications/consequences  Restrictions/precautions  Extent of focus on activity pre and post op  Information about procedure/resumption of activity  Certainty of timescales  Extent of consistent/tailored advice and support re RTW/RUA provided by clinicians/AHPs  Follow-up/post op rehab |
| Occupational intervention | What would an occupational intervention for patients look like? | Who would deliver? Individual/team/profession/expertise  When, how? Where? Ward – outpatient clinic, community  Format – paper, on-line, phone apps, one-to-one sessions  Components – assessment, advice, information, liaison, signposting, workplace visits |
| Outcome measurement | We are developing an intervention to help people RTW/RUA after surgery. How do you think we might best measure the effectiveness of the intervention from your perspective? | Functional performance  Duration of sick leave  Sustained RTW/RUA  Wellbeing |
|  | | |
| Is there anything else that you would like to say that we haven’t already discussed? | | |
