# Supplementary material for: Using intervention mapping to develop an occupational advice intervention to aid return to work following hip and knee replacement in the United Kingdom
Source: BMC Health Serv Res. 2020 Jun 9;20:523. doi: 10.1186/s12913-020-05375-3 (PMC7285551; doi:10.1186/s12913-020-05375-3)
Supplement: Supplementary file 3 — Additional file 3. Summary of the key information developed from IM Step 1, based on the PICO format. [file 12913_2020_5375_MOESM3_ESM.docx]

**Additional file 3. Summary of the key information developed from IM Step 1, based on the PICO format**

**POPULATION: The return to work population**

***The need for a return to work intervention***

- A substantial proportion (up to 25%) of patients are in work prior to surgery, including some past state pension age. **(COHORT)**
- A minority of patients have access to occupational health services and knowledge about employer sickness policies and sick pay is poor. **(COHORT AND INTERVIEWS)**
- A considerable proportion of patients return to work by 16 weeks, either to their usual job and/or amended hours and/or duties. **(COHORT)**
- While a significant proportion of patients might benefit from an occupational advice intervention, ‘standard care’ is currently sufficient to get the majority of patients back to work after surgery **(COHORT and INTERVIEWS)**

***Characteristics of the return to work population***

- Many patients are in full-time employment, in physically demanding roles and often at work until the day before surgery. **(COHORT)**
- Most patients need to drive, either to, or at, work. **(COHORT)**
- Patients often have a strong motivation to undergo joint replacement to improve their quality of life, reduce pain and continue work. **(INTERVIEWS)**
- Some patients are keen to return to work as soon as possible, sometimes too early (particularly the self-employed) and struggle to consider ‘lighter duties’. **(INTERVIEWS)**
- Only around a half of patients are provided with a fit note and most are given by the patient’s GP. The majority of fit notes prescribe the patients as ‘not fit for work’ for six weeks. **(COHORT)**

***Expectations of patients and healthcare teams***

- While some patients want to be fully fit before returning to work others are happy to return on a phased or amended return while they continued to recover. **(INTERVIEWS)**
- Patients expect to be off work for between two and three months after surgery. **(COHORT)**
- Many do not want to inconvenience their employer. **(INTERVIEWS)**
- There is an overall perception amongst clinicians that return to work is a realistic goal for the majority of patients undergoing joint replacement. However, expectations need to be managed carefully. **(INTERVIEWS)**

***Perceived barriers and facilitators to return to work***

- The motivation of the employee is a key factor in returning to work; and compliance with rehabilitation and self-management of health. **(INTERVIEWS)**
- Patients feel their employment status and need to remain in employment are not fully taken into consideration in the surgical decision-making process. **(INTERVIEWS)**
- Patients often do not consider the impact surgery will have on their ability to work until they are listed for surgery. **(INTERVIEWS)**
- Workplaces are generally able to accommodate patients’ needs for workplace adaptions and changes in working patterns. The majority of patients have some autonomy over how their work is planned. **(COHORT AND INTERVIEWS)**
- Office-based and non-manual work roles are considered easier to return to. The use of analgesia might preclude some work tasks. **(INTERVIEWS)**
- Employers concerns about health and safety and potential litigation might impact on return to work. **(INTERVIEWS)**
- Return to work planning prior to surgery by the employer is seen as a potential facilitator to return to work. **(INTERVIEWS)**
- Organisations with on-site occupational health are seen as having an advantage in supporting employees’ return to work following surgery. However there are concerns that occupational health might take an out-dated approach to recovery, might not have an in-depth knowledge of the patient’s job, or be risk-averse and extend sick leave unnecessarily. **(INTERVIEWS)**
- Surgery itself can be a barrier to returning to work, including postoperative symptoms of pain, oedema, low mood and fatigue, and restrictions such as hip precautions. **(INTERVIEWS)**

**INVERVENTION: An Occupational Advice Intervention**

***Perceived need***

- There is widespread workplace support for an occupational advice intervention, for use by employees and employers. **(INTERVIEWS and SURVEY)**
- Currently employers are reliant on employee feedback, and are not necessarily aware of the content of the information patients receive until the employee has returned to work. Having a standardised, approved intervention is seen as potentially beneficial. **(INTERVIEWS)**
- Accurate information about expected recovery might encourage patients to have surgery earlier, and prompt patients to discuss the timing of surgery with their employer, which could benefit their future health and work prospects. **(INTERVIEWS)**
- Providing occupational advice is not seen as a priority compared with addressing other patient needs. The intervention might require increased resources, and existing service level agreements might limit the extent of support available from the hospital orthopaedic team. **(INTERVIEWS AND SURVEY)**
- An occupational advice intervention to establish that a) helps those who would have returned to work using standard care get back to work earlier; b) helps improve rates of full sustained return to work or; c) helps those patients who would not have returned to work using standard care get back to work is desirable as they would produce overall benefits to the patient, employer and society all are desirable. **(EVIDENCE SYNTHESIS and INTERVIEWS)**
- Any intervention should complement rather than replace existing pre-operative information. Most trusts have invested significant time and resource creating patient resources and the occupational advice intervention should sit alongside these. **(INTERVIEW and SURVEY)**
- Delivering a tailored intervention for individual patients is considered difficult in practice, but potentially of value to the NHS and to employers. **(INTERVIEWS)**

**Content and format**

- An individualised or personalised approach to the intervention might be required because of the differences in employee characteristics and circumstances. **(INTERVIEWS)**
- Suggestions for the content of the intervention include information about occupational health, complications, restrictions, signposting, advice lines, symptom management and information on expected recovery milestones, as well as advice regarding sick-notes, negotiating modifications, and medication and work. Advice should include the psychosocial impacts of returning to work, such as feelings of isolation, fatigue, loss of identity and confidence, and anxiety. **(INTERVIEWS)**
- Generic components of previously developed return to work interventions include: work simulation, work hardening and job simulation; contact with the employers; physical therapy and exercise; educational content; vocational counselling and guidance; multidisciplinary team involvement. **(EVIDENCE SYNTHESIS)**
- A printed format for information materials is favoured by patients, health practitioners and employers. Many are positive about using digital resources; however computer literacy does not mean that patients would prefer their advice exclusively by this method. **(INTERVIEWS)**
- Employers favour information in a paper format that other stakeholders can access and contribute to at different stages in the process of return to work that would aid clarity and transparency of information. **(INTERVIEWS)**

**Delivery and Timing**

- Some patients might not be able or willing to process a great deal of written one-way information, and a more personal verbal approach – group and individual, face-to-face and by phone - has advantages in terms of opportunities for asking questions and for seeking clarification and explanation. **(INTERVIEWS)**
- Some GPs suggest using a format similar to that used for new parents (the Red Book) that other stakeholders could use and contribute to. **(INTERVIEWS)**
- Employers are keen to be recipients of the intervention. There is support for designing the intervention in such a way that the employers can see or be provided with information about the operation, and a copy of any generic and individual work-related advice, rather than simply relying only on the patient to report that information. Employers also suggest that the intervention include information for employees as to how they can help themselves at different stages in the surgical pathway. **(INTERVIEWS)**
- There are differing views regarding who should be delivering the intervention and the timing of the intervention. **(INTERVIEWS)**
- Evidence indicates that healthcare-based return to work rehabilitation is best delivered by multi-disciplinary teams using a biopsychosocial approach and a tailored ‘stepped care’ model. **(EVIDENCE SYNTHESIS)**
- Current NHS resources are seen as a barrier to the advice and support available to those returning to work, because clinicians have less time to spend with individual patients, including post-operative physiotherapy. **(INTERVIEWS)**

**COMPARATOR: Advice currently provided to return to work patients**

**Current delivery of return to work advice**

- Patients currently receive a range of written advice and information in a variety of formats from secondary care prior to surgery. However, the advice received does not usually include information about return to work, and tends to focus on the needs of an older retired population. **(INTERVIEWS and SURVEY)**
- The delivery of occupational advice is not generally seen as the role of, or a priority for, the orthopaedic team. There is a perceived dependence on doctors to guide the recovery process and some of this responsibility could be delegated to other healthcare professionals. **(INTERVIEWS and SURVEY)**
- Occupational advice is generally given ad-hoc, verbally, and at patient request. **(SURVEY)**
- Most clinicians have only a superficial understanding of any occupational advice provided to patients through the hospital orthopaedic team. **(INTERVIEWS and SURVEY)**
- Many hospital orthopaedic staff feel unable to provide advice about returning to work and most AHPs take their lead from, or defer, to the surgeon. **(INTERVIEWS and SURVEY)**
- Surgeons feel they lack the necessary knowledge of patients’ occupations, and the skills to give more than general advice. **(INTERVIEWS)**
- Surgeons frequently refer to return to work advice in terms of ‘blanket’ timescales - often six and twelve weeks of sickness absence - which usually coincides with follow-up appointments and advice on returning to drive. These timescales appear mainly to be used for the sake of convenience, with some basis in clinical reasoning and experience. **(INTERVIEWS and SURVEY)**
- Surgeons’ advice tends to focus on whether or not someone would eventually return to their job, and how long they might be on sick leave, rather than rehabilitation ‘on the job’ through adjustments. **(INTERVIEWS)**

**Structure of current NHS services**

- The structure of existing pre-admission and pre-operative education programmes is extremely varied both in terms of content, timing and the healthcare team members delivering this information. Resources are at a premium therefore any occupational advice intervention should be embeddable within existing pathways without the need for significant service restructure. An occupational advice intervention therefore needs to be pragmatic and deliverable within current healthcare settings **(INTERVIEWS and SURVEY)**
- Most surgeons do not see their patients again after listing for surgery until the day of surgery and then only once after surgery limiting the opportunities to discuss return to work issues. **(INTERVIEWS and SURVEY)**
- Communication with other stakeholders about patients’ return to work or other occupational matters is limited. Patients are the main conduit of information and advice for employers, which depends on how the patient interprets and communicates the advice given by the surgeon. Clinic/discharge letters to the GP rarely focuses on work issues. **(INTERVIEWS)**
- GPs see their main role as supporting (and not ‘interfering’ with) the medical treatment of the patient after surgery. They assume the main responsibility for advising on work to rest with the hospital team and/or physiotherapists, or with occupational health departments. GPs report that their role with patients is restricted by lack of resources, particularly time, and their skillset. Employers perceived that GPs are variable in the support they provided in return to work, and inclined to be overcautious. **(INTERVIEWS)**
- The opportunity for patients to receive advice/information from AHPs and nurses varies between trusts. In some cases separate preoperative group education classes are held for hip and knee patients, in others these are combined. **(INTERVIEWS and SURVEY)**
- Occupational therapy is generally not routinely provided for knee replacement patients, particularly those under 60 years old, because they rarely need adaptive equipment on discharge. **(INTERVIEWS)**
- Post-operative physiotherapy for knee patients however is favoured. Hip patients are unlikely to receive physiotherapy post-operatively as routine, although individual patients might be referred depending on need. **(INTERVIEWS)**

**OUTCOME: Measurement of return to work**

**How is return to work measured?**

- There is no standardised method of measuring ‘return to work’. **(EVIDENCE SYNTHESIS)**
- A variety of tools have previously been used to assess return to work after surgery or for musculoskeletal conditions. Generally measures used in the literature fall in to one of the following categories: non-standardised return to work/activities measures, standardised scales for return to work/usual activities, measures focusing on musculoskeletal symptoms, quality of life, psychological and other measures. Number of days of sick leave is also commonly used. Patient reported outcome measures tend to focus more broadly on activities of daily living. **(EVIDENCE SYNTHESIS)**
- Other potential measures might include retention/relapse following return to work, sickness absence prior to surgery, work ability/ performance, use of analgesia, and whether expectations of surgery/return to work are met. **(INTERVIEWS and SURVEY)**
- As regards measuring the impact of the intervention potential measures included qualitative assessment of the process and the extent to which any intervention was accessed and perceived to be useful. **(INTERVIEWS)**
