# Supplementary material for: Using intervention mapping to develop an occupational advice intervention to aid return to work following hip and knee replacement in the United Kingdom
Source: BMC Health Serv Res. 2020 Jun 9;20:523. doi: 10.1186/s12913-020-05375-3 (PMC7285551; doi:10.1186/s12913-020-05375-3)
Supplement: Supplementary file 4 — Additional file 4. Preliminary list of patient performance objectives. [file 12913_2020_5375_MOESM4_ESM.docx]

**Additional file 4. Preliminary list of patient performance objectives**

| **Patient Performance Objectives** | | **Stage in pathway** | **Examples of unresolved questions?** |
| --- | --- | --- | --- |
| 1. | Patient makes informed decision about surgery with respect to work | At/following first clinic appt | How will this be done? Whose responsibility is it to enable this? What is the role of the GP / Surgeon? |
| 2. | Patient is provided with advice and information about recovery and RTW | Following first clinic appt/listing | What information is important? How and when will the information be delivered? |
| 3. | Patient provides employer with accurate information about their planned surgery and recovery | Prior to surgery | What information will the employers receive? How will this be delivered to employers? |
| 4. | Patient completes RTW checklist detailing their work demands (with employer as required) | Prior to surgery | What information will be included in the checklist? When will it be completed? |
| 5. | Patient identifies and prioritises potential barriers and solutions to a safe and appropriate RTW | Prior to surgery | How will patients do this? Will they do this with their employer? What skills to we need to equip them with to allow this to happen? |
| 6. | Patient engages with hospital team allowing pre-operative development of a RTW plan | Prior to surgery | Which member of the hospital orthopaedic team and when will this happen? What training will be involved? |
| 7. | Patient meets with their employer to discuss their recovery and provisional RTW plan | Prior to surgery | How long before surgery will this happen? Will it happen after the employer has received the information in PO3? |
| 8. | ‘At risk’ patient engages in a minimum of three pre-operative follow-ups (phone calls/meet ups) with member of hospital staff to help develop a RTW plan and enable safe RTW | Prior to surgery | How do we identify ‘at risk patients’? Is it feasible to offer 3 pre-operative appointments? What are the resource implications? Will patients be able to attend if they are continuing to work? |
| 9. | Patient communicates with employer regarding surgical outcome and progress/recovery | Following surgery | How soon after surgery should they do this? How will the patient / employer get information about the post-operative recovery? |
| 10. | Patient revises RTW plan following surgery as necessary with their employer and hospital staff | Following surgery | How will this happen (especially is patient not routinely followed up / offered post-operative therapy)? |
| 11. | ‘At risk’ patient engages in a minimum of three post-operative follow-ups (phone calls/meet ups) with member of hospital staff to  Review progress with RTW plan | Following surgery | How do we identify ‘at risk patients’? Is it feasible to offer 3 pre-operative appointments? What are the resource implications? |
| 12. | Patient adheres to postoperative rehabilitation plan and advice | Following surgery | Can we monitor this? How do we ensure it happens? |
| 13. | Patient seeks help and support regarding RTW as required postoperatively | Following surgery | How do we facilitate this? What is the mechanism for support? |
