# Supplementary material for: Using intervention mapping to develop an occupational advice intervention to aid return to work following hip and knee replacement in the United Kingdom
Source: BMC Health Serv Res. 2020 Jun 9;20:523. doi: 10.1186/s12913-020-05375-3 (PMC7285551; doi:10.1186/s12913-020-05375-3)
Supplement: Supplementary file 5 — Additional file 5. Preliminary list of staff performance objectives. [file 12913_2020_5375_MOESM5_ESM.docx]

**Additional file 5. Preliminary list of Hospital Orthopaedic Team staff performance objectives**

| **Staff performance objectives** | | **Stage in pathway** | **Examples of unresolved questions** |
| --- | --- | --- | --- |
|  | |  |  |
| 1 | Staff screen patients that intend to RTW prior to meeting with surgeon | At first clinic appt/listing | How will this be done? Which team member will be responsible? |
| 2 | Surgeon asks patients about their usual work and expectations of RTW following surgery | At first clinic appt/listing | How do we ensure this is done? What tools can we develop to enable this process? |
| 3 | Surgeon discusses pros and cons of surgery with patient including timescales of surgery - in relation to patients’ usual work | At first clinic appt/listing | Need to train and empower surgeons - how do we get them to engage? |
| 4 | Surgeon considers patients’ work schedules when listing them for surgery | At listing | How do they get this information and is this possible? |
| 5 | Staff screen patients to identify those who are perceived to be at risk of not making a safe and appropriate/expected RTW | At listing | How do we identify ‘at risk’ patients and what tools could assist with this? |
| 6 | Staff provide all patients with RTW advice manual and contact phone/email | At listing | What will the patient manual include? |
| 7 | Staff provide patients with generic written information relating to surgery/RTW to give to their employer/colleagues | At listing | What will the employer manual include? |
| 8 | Staff provide ‘at risk’ patients with RTW checklist to complete with their employer if necessary (i.e. if patient unable to answer questions about availability of modified work) | At listing | How do we identify ‘at risk’ patients and what tools could assist with this? What would the checklist include? |
| 9 | Staff make a minimum of three pre-operative follow-ups (phone calls/meet ups) with patients in ‘at risk’ group to:  Review occupational checklist  Identify potential barriers and solutions to safe and appropriate RTW  Develop a RTW plan  Liaise with employer as appropriate | Pre-op | Is this possible? What are the resource implications of 3 pre-operative interactions? Will patients have time for this and be willing to engage with it? |
| 10 | Staff routinely include the topic of RTW in group pre-op education and identify any ‘at risk’ patients to… (as per PO9) | Pre-op | How do we signpost RTW patients to the pre-op education team? What information do they need to cover? |
| 11 | Staff routinely ask patients at pre-assessment about RTW and identify any ‘at risk’ patients to… (as per PO9) | Pre-op | How do we signpost RTW patients to the pre-assessment teams? What information do they need to cover? |
| 12 | Surgeon liaises with treatment team regarding patient’s post-op recovery and how this may impact on patient’s RTW | Post-op prior to discharge | Will surgeons take an active interest? |
| 13 | Staff complete a post-operative screening tool to identify ‘at risk’ patients | Post-op prior to discharge | When will this be done and who will do it? How do we identify ‘at risk’ patients and what tools could assist with this? |
| 14 | Staff advise on revision of the patient’s RTW plan as necessary following surgery | Post-op | Which staff and when will this happen? |
| 15 | Staff summarise patient’s expected RTW outcome and RTW plan in ward discharge letter | Post-op | How will junior doctors on the ward find this information? What specific information will be sent to the GP? |
| 16 | Staff give a copy of the ward discharge letter to the patient addressed to their employer to pass on if they wish to | Post-op | Who will do this? Will patients be happy to share this information with their employers |
| 17 | Staff ask each patient whether they require a fit note on discharge | Post-op prior to discharge |  |
| 18 | Staff complete fit notes in accordance with best practice guidelines and hospital standard contract | Post-op prior to discharge | How do we determine the duration of the fit note and what recommendations for work are included? |
| 19 | Staff offer all RTW patients a minimum of three post-op physiotherapy/rehabilitation appointments | Post-op | Is this feasible? (not routine care for all) |
| 20 | Staff offer all ‘at risk’ patients a minimum of six post-op physiotherapy/rehabilitation appointments | Post-op | Is this feasible? (not routine care for all), How do we identify ‘at risk’ patients for this more intensive approach |
| 21 | Staff conduct a minimum of three follow-up phone calls/meet ups with ‘at risk’ patients to review progress with RTW plan, support/signpost, liaise with employer as required | Post-op | Is this feasible? (not routine care for all), how much staff time will be required? Are the resources available for this? |
| 22 | Staff summarise and record patient’s RTW status/outcome in all out-patient clinic notes and following each appointment with therapists | Follow-up | When will they do this and where will they record the information so that it is visible? |
| 23 | Staff discharge patient from the orthopaedic service when the patient has RTW | Discharge | Some people may not return to work so this implies they will remain under orthopaedics – for how long should orthopaedic teams offer follow up for RTW issues? |
