# Supplementary material for: Using intervention mapping to develop an occupational advice intervention to aid return to work following hip and knee replacement in the United Kingdom
Source: BMC Health Serv Res. 2020 Jun 9;20:523. doi: 10.1186/s12913-020-05375-3 (PMC7285551; doi:10.1186/s12913-020-05375-3)
Supplement: Supplementary file 6 — Additional file 6. Parameters, methods and practical applications for patient determinants [file 12913_2020_5375_MOESM6_ESM.docx]

**Additional file 6. Parameters, methods and practical application for patient determinants**

| **KNOWLEDGE/AWARENESS** | **Methods** | **Definition** | **Parameters** | **Applications** |
| --- | --- | --- | --- | --- |
| Aware that completing an occupational checklist in clinic will inform the surgeon about their work activities and demands | Consciousness raising *(Health Belief Model)* | Providing information about the consequences for a problem behaviour | Raising awareness must be quickly followed by increase in problem solving ability | *Outpatient clinic staff* explain that a completed checklist will help prompt the surgeon and the patient to discuss work issues in full otherwise they might not make the optimum decision about surgery |
| Knows the risks/benefits of surgery and RTW rates and likely impact of surgery on their ability to do their job | Personalise risk *(Precaution Adoption Process Model)* | Provide information about personal costs or risks of action or inaction with respect to target behaviour | Present messages as individual and undeniable | *Individuals receive personal risk feedback from surgeon* on potential RTW outcomes in relation to their work situation (*surgeon prompted about potential risks by referring to patient’s occupational checklist)* |
| Knows key advice and information concerning recovery and RTW e.g.  Work modifications  Fit notes  Restrictions  Milestones  Sick leave | Coherence and imagery *(Theories of Information Processing)*  Discussion & elaboration *(Elaboration Likelihood Model)*  Reinforcement *(Learning Theories)* | Encourage consideration of a topic in open informal debate  Linking a behaviour to any consequence that increases it | Listening to the learner to ensure that the correct schemas are activated | A RTW workbook is provided to patient *by* *outpatient clinic staff* containing advice and information – sections of text have logical order and clearly related to each other, use graphical representations  Contents and use of RTW workbook are referred to in discussions with *all members of HOT: surgeon, RTWC, preoperative education/presentation, ward staff, outpatient therapy staff* |
| Aware that bringing the RTW workbook to each appointment is expected in order to encourage patients and staff focus on RTW  Aware that discussing the content of the RTW workbook with hospital staff is expected to encourage patients and staff to focus on RTW | Personalise risk *(Precaution Adoption Process Model)*  Reinforcement *(Learning Theories)* | Provide information about personal costs or risks of action or inaction with respect to target behaviour  Linking a behaviour to any consequence that increases the behaviour’s rate, frequency or probability | Present messages as individual and undeniable  Needs to be tailored to the individual, group or organization, follow the behaviour in time, and seen as a consequence of the behaviour | *Outpatient clinic staff inform patient* that they are expected to bring the RTW workbook to enable the HOT advise them on their individual RTW. *Outpatient clinic staff draw attention to this instruction in the workbook*  Members of the HOT (*pre-op assessment and education teams, RTWC, ward staff, therapy teams*) ask patients if they have brought their RTW workbook to each appointment; praise patients for bringing their RTW workbook to each appointment; discuss the content of the patient’s RTW workbook at each appointment |
| Can describe how to assess the demands of their work and set an approximate RTW date, and how to do this with their employer if required | Modelling *(Social Cognitive Theory)*  Variety of media *(Theories of Information Processing)* (repeated exposure)  Elaboration *(Theories of Information Processing)* | Providing an appropriate model  Stimulating the learner to add meaning to the information that is processed | Identification with the model, model receives positive reinforcement, coping vs. mastery model  Messages that are personally relevant | Examples of other patients’ work demands and setting approximate RTW dates *included in workbook/on website* and at *preoperative presentations given by staff*  Discussions with *RTWC* and *preoperative education and assessment teams* |
| Can list the potential barriers and solutions to their own RTW and develop a RTW plan, with employer as required | Modelling *(Social Cognitive Theory)*  Variety of media *(Theories of Information Processing)*  Elaboration *(Theories of Information Processing)* | Providing an appropriate model  Stimulating the learner to add meaning to the information that is processed | Identification with the model receives positive reinforcement, coping vs. mastery model  Messages that are personally relevant | Examples of other patients’ barriers and solutions and RTW plans *included in workbook/on website* and *at preoperative presentations given by staff*  Discussions with *RTWC* and *preoperative education and assessment team* |
| Can describe the process of engaging with their RTWC by phone or face-to-face at the hospital to further develop their RTW plan (how, when, where) | Variety of media Mere/repeated exposure *(Theories of Information Processing)* |  |  | Information about engaging with the RTWC is given verbally by *outpatient clinic staff*, in *the patient workbook* and on *website*, on *discharge letter* -and posters on the ward? |
| Know what information to provide to their employer* /workplace, and who should receive it | Modelling *(Social Cognitive Theory)*  Discussion *(Elaboration Likelihood Model)* | Providing an appropriate model  Encourage consideration of topic in open informal debate | Identification with the model  Listening to the learner to ensure that the correct schemas are activated | Examples of other patients’ negotiation w employer  *Outpatient clinic staff* explain that giving information to their employer will help the employer to understand their surgery and to help them plan the patient’s RTW.  *Outpatient clinic staff* will suggest the individuals in the workplace who might best receive the employer information. |
| Know the likely impact of surgery on their RTW and how to discuss their RTW with their employer* prior to surgery | Discussion *(Elaboration Likelihood Model)* | Encourage consideration of topic in open informal debate | Listening to the learner to ensure that the correct schemas are activated | *Discussion with/coaching by RTWC* |
| Know why a RTW plan may need to be revised following surgery and how to do this | Scenario-based risk information  *(Precaution Adoption Process Model)* | Providing information that may aid the construction of an image of the ways in which a future loss or accident might occur | Plausible scenario with a cause and scenario | *Discussions with surgeon, RTWC, ward staff, outpatient therapy staff* regarding unexpected outcomes of surgery and how these might impact on their RTW and RTW plan |
| Know the process of engaging with the RTWC via the RTW helpline following surgery (who, when, how, what to expect) | Variety of media Mere/repeated exposure *(Theories of Information Processing)* |  |  | Information is provided verbally *by RTWC, ward staff, outpatient clinic* staff, in writing in the *patient workbook and on website,* on *discharge letter -and posters* on the ward and in clinic? |
| Know their postoperative rehabilitation plan and the risks of not adhering to it | Scenario-based risk information  *(Precaution Adoption Process Model)* | Providing information that may aid the construction of an image of the ways in which a future loss or accident might occur | Plausible scenario with a cause and scenario | *Discussions with: surgeon, RTWC, pre-op education/presentation, ward staff, outpatient therapy staff* – about the pros and cons of not adhering to their rehabilitation plan |
| **SELF-EFFICACY/SKILLS** | **Methods** | **Definition** | **Parameters** | **Application** |
| Able to complete an occupational checklist prior to appointment with surgeon | Verbal persuasion *(Social Cognitive Theory)*  Facilitation *(Social Cognitive Theory)* | Using messages that suggest the participant possesses certain qualities  Creating an environment that reduces barriers to action | Credible source  Requires identification and removal of barriers | *Outpatient clinic staff* explain that this is an activity that they believe the patient can do  Sufficient checklists available, clipboard, pens, time to complete, actual help provided by *outpatient clinic staff* |
| Able to process information about surgical procedure and make informed choice | Motivational interviewing *(Self-Determination Theory)*  Individualisation *(Trans Theoretical Model)* | Collaborative goal-orientated style of communication  Provide opportunities for learners to have personal questions answered or paced according to progress | Must recognize collaboration, exploration, autonomy  Personal communication that responds to a learner’s needs | *Surgeon supports autonomy of patient in consultation* by valuing patient perspective, offering choices, minimizing pressures  *Surgeon facilitates communication* at consultation  *RTWC, pre-op assessment and education teams* provide further opportunities to discuss decision with RTW following consultation |
| Can acquaint themselves with key information about recovery and RTW provided in the RTW workbook | Verbal persuasion *(Social Cognitive Theory)*  Goal-setting (GST, *(Theories of Self-Regulation)* | Using messages that suggest the participant possesses certain qualities  Prompting the patient to plan what they will do to reach the target behaviour | Credible source  Patient’s commitment to the goal | *Outpatient clinic staff* and *RTWC* explain that the workbook has been designed for and approved by patients.  *Outpatient clinic staff* and *RTWC* discuss and agree the goal for the next appointment (e.g. to read/complete a particular section of workbook) |
| Can bring the RTW workbook to each hospital appointment  Can discuss their RTW workbook with hospital staff | Verbal persuasion *(Social Cognitive Theory)*  Planning coping responses *(Theories of Self-Regulation)*  Guided practice *(Social Cognitive Theory)* | Using messages that suggest the participant possesses certain qualities  Prompting patients to list potential barriers and ways to overcome these  Prompting individuals to rehearse and repeat the behaviour various times, discuss the experience, and provide feedback | Credible source  Identification of barriers and practice coping response  Requires supervision by an experienced person | *Outpatient clinic staff* and *RTWC* explains that this is an activity that they believe patients can do  *Outpatient clinic staff* and *RTWC* discuss potential reasons why workbook might not be brought to appointment and formulate solutions with patient  *RTWC* models target behaviour a number of times, then asks patient to do the same and gives comments, emphasizing what has been done well |
| Can complete the sections of the RTW workbook that will help them understand the demands of their work and set an approximate RTW date  (with employer* if required) | Modelling *(Social Cognitive Theory)*  Planning coping responses *(Theories of Self-Regulation)* | Providing an appropriate model  Prompting patients to list potential barriers and ways to overcome these | Identification with the model  Able to identify barriers and practice coping response | Examples of other patients’ job demands *in workbook/on website*, also shared *by RTWC* and *at preoperative presentations given by staff*  *Outpatient clinic staff* and *RTWC* discuss potential reasons why patients might struggle to discuss demands with employer, and formulate solutions with patient |
| Can identify barriers/ facilitators to their own safe and appropriate RTW and develop a RTW plan (with employer if required) | Modelling *(Social Cognitive Theory)*  Planning coping responses *(Theories of Self-Regulation)* | Providing an appropriate model  Prompting patients to list potential barriers and ways to overcome these | Identification with the model  Able to identify barriers and practice coping response | Examples of other patients’ barriers and solutions and RTW plans *in workbook/on website*, also *shared by RTWC and at preoperative presentations given by staff*  *Outpatient clinic staff* and *RTWC* discuss potential reasons why patients might struggle to identify barriers/facilitators with employer, and formulate solutions with patient |
| Can engage with the RTWC to further develop their RTW plan – minimum of one contact | Individualisation | Providing opportunities to have personal questions answered or instructions paced according to individual progress | Personal communication that responds to an individual’s needs | *RTWC contacts patient* to help them develop their own individual RTW plan |
| Can provide written information provided by the HOT about their planned surgery and recovery/RTW advice to their employer*/workplace | Verbal persuasion *(Social Cognitive Theory)* | Using messages that suggest the participant possesses certain qualities | Credible source | *Outpatient clinic staff and RTWC* explain that this is an activity that they believe patients can do  *Outpatient clinic staff* give each patient an information booklet to give to their employer |
| Can meet with their employer* to discuss their recovery and RTW plan | Verbal persuasion *(Social Cognitive Theory)*  Planning coping responses *(Theories of Self-Regulation)*  Implementation intentions *(Goal Setting Theory)* | Using messages that suggest the participant possesses certain qualities  Prompting patients to list potential barriers and ways to overcome these  Making plans for any obstacles that occur | Credible source  Identification of barriers and practice coping response | *Outpatient clinic staff* and *RTWC* explains that this is an activity that they believe patients can do  *Outpatient clinic staff/RTWC* discuss potential causes and formulate solutions with patient  *RTWC* helps patient to prepare an If…Then plan ready if they encounter any difficulties with their employer |
| Communicate with their employer regarding their surgical outcome and progress/recovery | Guided practice *(Social Cognitive Theory)* | Prompting individuals to rehearse and repeat the behaviour various times, discuss the experience, and provide feedback | Requires supervision by an experienced person | *RTWC* models target behaviour a number of times, then asks patient to do the same and gives comments, emphasizing what has been done well |
| Negotiate a revised RTW plan with their employer and RTWC if necessary | Verbal persuasion *(Social Cognitive Theory)*  Planning coping responses *(Theories of Self-Regulation)*  Guided practice *(Social Cognitive Theory)*  Modelling *(Social Cognitive Theory)* | Using messages that suggest the participant possesses certain qualities  Prompting patients to list potential barriers and ways to overcome these  Prompting individuals to rehearse and repeat the behaviour various times, discuss the experience, and provide feedback | Credible source  Identification of barriers and practice coping response  Requires supervision by an experienced person | *RTWC* explains that this is an activity that they believe patients can do  *RTWC* formulates solutions with patient  *RTWC* models target behaviour a number of times, then asks patient to do the same and gives comments, emphasizing what has been done well |
| Ability to engage with RTWC via helpline if they are having problems post discharge | Planning coping responses *(Theories of Self-Regulation)* | Prompting patients to list potential barriers and ways to overcome these | Identification of barriers and practice coping response | *RTWC* formulates solutions with patient to overcome any barriers they might experience in using the helpline |
| adhere to their postoperative rehabilitation plan  attend/travel to rehabilitation if required | Planning coping responses *(Theories of Self-Regulation)*  Facilitation  *(Social Cognitive Theory)* | Prompting patients to list potential barriers and ways to overcome these  Creating an environment that makes the action easier or reduces barriers to action | Identification of difficult situations and practice of coping response  Requires identification and removal of barriers | *RTWC* formulates solutions with patient  *RTWC* asks patient the optimum arrangements for any rehabilitation they require and liaises with outpatient therapy teams |
| **ATTITUDES, BELIEFS, EXPECTATIONS** | **Methods** | **Definition** | **Parameters** | **Application** |
| Believes that completing an occupational checklist will facilitate RTW | Information about others approval | Providing information about what others think about the persons behaviour | Positive expectations available in the environment | *Outpatient clinic staff* inform patient that the surgeon will approve of them completing the checklist |
| Is willing to take responsibility for surgical decision | Motivational interviewing | Explore persons reasons for change within atmosphere of acceptance | Supportive relationship between client and professional | *Surgeon supports* autonomy of patient and offers choices about surgery where possible |
| Has realistic expectation of RTW outcome following surgery | Individualisation | Providing opportunities for learners to have personal questions answered | Personal communication that responds to a learner’s needs | *Surgeon* advises individual patient as to the likely outcome of RTW following surgery according to the patient’s characteristics and work demands |
| Believes that having a good understanding about recovery and RTW through RTW workbook is likely to lead to a positive RTW outcome | Persuasive communication  Repeated exposure | Guiding individual toward adoption of action by using arguments and other means  Making a stimulus repeatedly accessible to the individuals sensory receptors | Messages need to be relevant and not too discrepant to beliefs of individual | HOT – especially *surgeon, outpatient clinic staff, RTWC* all present positive attitude to use of RTW workbook  *All members of the team* consistently refer to intervention. Posters on ward? |
| Believes that bringing the RTW workbook to each hospital appointment is likely to facilitate a positive RTW outcome  Believes that them discussing the RTW workbook at each hospital appointment is likely to facilitate a positive RTW outcome | Persuasive communication  Anticipated regret | Guiding individual toward adoption of action by using arguments and other means  Stimulate people to focus on their feelings after unintended risky behaviour | Messages need to be relevant and not too discrepant to beliefs of individual  Stimulation of imagery; assumes a positive intention to avoid the risky behaviour | HOT – especially *surgeon, outpatient clinic staff, RTWC* refer to example of Red Book given to new parents as an example of similar approach in healthcare, and importance of good communication  *Outpatient clinic staff, surgeon, RTWC* asks individual to imagine what might happen if they did not bring the RTW workbook to each appointment |
| Expects that completing the sections of the workbook that will help them understand the demands of their work and set an approximate RTW date…..with their employer* if required is likely to lead to a positive RTW outcome | Framing *(Health Belief Model)*  Modelling *(Social Cognitive Theory)* | Using gain-framed messages emphasizing the advantages of performing the healthy behaviour –  Or loss-framed messages  Providing an appropriate model | Requires high self-efficacy expectations  Identification with the model | *Outpatient clinic staff, surgeon, RTWC* and the *RTW workbook* emphasise the advantages of completing the workbook  *Outpatient clinic staff, surgeon, RTWC* and the *RTW workbook* provide examples – coping models |
| Believes that identifying barriers/facilitators and developing a RTW plan will aid their own safe and appropriate RTW | Modelling *(Social Cognitive Theory)*  Framing *(Health Belief Model)* | Providing an appropriate model  Using gain-framed messages emphasizing the advantages of performing the healthy behaviour –  Or loss-framed messages | Identification with the model  Requires high self-efficacy expectations | *Outpatient clinic staff, surgeon, RTWC* and the *RTW workbook* provide examples – coping models  *Outpatient clinic staff, surgeon, RTWC and the RTW workbook* emphasise the advantages of completing the workbook |
| Believes that engaging with the RTWC and developing a RTW plan will lead to a positive RTW outcome | Individualisation  Elaboration *(Elaboration Likelihood Model)* | Providing opportunities for learners to have personal questions answered  Stimulating the learner to add meaning to information that is processed | Personal communication that responds to a learner’s needs  Messages that are personally relevant | *RTWC* advises and supports individual patient with their RTW plan according to their individual characteristics and work demands  *RTWC* discusses the RTW plan with the individual patient |
| Believes that providing their employer with written information about their forthcoming surgery and RTW will facilitate their RTW. | Consciousness raising  *(Health Belief Model)* | Providing information about causes, consequences, alternatives | Can use feedback and confrontation, but raising awareness must be quickly followed by increase in self-efficacy | Key people in HOT e.g*. surgeon, outpatient clinic staff, RTWC, and workbook* provide patient with feedback from Phase 1 of the study where employers stated they would like more information about surgery and recovery to help employees RTW |
| Believes that meeting with their employer informing their employer* to discuss their recovery and RTW plan will facilitate their RTW. | Belief selection  (TPB, RAA) | Using messages designed to strengthen positive beliefs, weaken negative beliefs and introduce new beliefs | Requires investigation of the individual’s current beliefs | *RTWC* explores patient’s beliefs when engaging with patient |
| Believes that communicating with their employer* regarding surgical outcome and progress will lead to a positive RTW outcome | Self re-evaluation | Encouraging combining both cognitive and affective assessments of one’s self-image with and without required behaviour | Needs stimulation of both cognitive and affective assessments of one’s self-image | *RTWC* encourages patient to compare his or her image as a person who does/does not communicate with their employer |
| Believes that revising the RTW plan following surgery will provide a more positive RTW experience | Modelling *(Social Cognitive Theory)*  Framing | Providing an appropriate model  Using gain-framed messages emphasizing the advantages of performing the healthy behaviour –  Or loss-framed messages | Identification with the model  Requires high self-efficacy expectations | *RTWC* and workbook/website provides examples of how patients have revised RTW plans following surgery |
| Believes that engaging with the RTWC via the RTW helpline/answering service will potentially alleviate any RTW problems | Modelling *(Social Cognitive Theory)* | Providing an appropriate model | Identification with the model | *Workbook/website* provides examples of how patients have contacted the RTWC via the helpline post-surgery |
| Believes that adhering to their postoperative rehabilitation plan is important for their recovery/RTW | Persuasive communication | Guiding individuals toward the adoption of an idea by using arguments or other means | Messages need to be relevant and not too discrepant from beliefs of individual | RTWC  RTW workbook and website |
| **PERCEIVED NORMS** | **Methods** | **Definition** | **Parameters** | **Application** |
| Recognises that nowadays patients are being encouraged to take an active part in their care | Anticipated regret | Stimulate people to focus on their feelings after unintended risky behaviour | Stimulation of imagery; assumes a positive intention to avoid the risky behaviour | *Outpatient clinic staff, surgeon, RTWC* asks individual to imagine how they would feel/what might happen if they did not make an informed decision about surgery |
| Recognises that RTW is now considered a health outcome and that this is a good thing | Consciousness raising  *(Health Belief Model)* | Providing information about causes, consequences, alternatives | Can use feedback and confrontation, but raising awareness must be quickly followed by increase in self-efficacy | Information in *RTW workbook, website* and *members of HOT* consistent in expressing their belief in work as a health outcome  Evidence about relationship between work and good health in *RTW workbook* |
| Perceives that it is usual for patients to make an informed decision about surgery with respect to their work | Shifting perspectives *(Theories of Stigma and Discrimination)* | Encourage taking the perspective of the other | Initiation from the perspective of the learner; needs imaginary competence | HOT enable patients to compare the potential result for patients who do, versus those who don’t make informed decision |
| Recognises that patients undergoing surgery acquaint themselves with key information about recovery and RTW provided by the hospital orthopaedic team  Recognises that discussing the RTW workbook with hospital staff is best practice | Information about others’ approval  Persuasive communication | Providing information about what others think about the persons behaviour  Guiding individuals toward the adoption of an idea by using arguments or other means | Positive expectations are available in the environment  Messages need to be relevant and not too discrepant from beliefs of individual | Patients are given information in their RTW workbook, and staff express approval of patients who acquaint themselves with key information about recovery and RTW provided by the hospital orthopaedic team  RTW workbook and website states that content informed by patients and other stakeholders and current evidence |
| Recognises that employers* are key stakeholders in RTW and involving them at an early stage can facilitate RTW | Consciousness raising  *(Health Belief Model)* | Providing information about causes, consequences, alternatives | Can use feedback and confrontation, but raising awareness must be quickly followed by increase in self-efficacy | Information in *RTW workbook, website* and *members of HOT* consistent in expressing their belief in involving employers at an early stage  Evidence about early involvement of employers in *RTW workbook* |
| Recognises that the ideal RTW process relies on coordination and joint planning between healthcare, the patient and their employer | Elaboration *(Elaboration Likelihood Model)* | Stimulating the learner to add information that is processed | Messages that are personally relevant, easily understandable | *RTWC and HOT* (e.g. pre-assessment education) encourage discussion of communication pathways |
| Recognises that employers do not necessarily know about this type of surgery and how best to facilitate RTW | Shifting perspectives *(Theories of Stigma and Discrimination)* | Encourage taking the perspective of the other | Initiation from the perspective of the learner; needs imaginary competence | HOT help patient to see RTW from the employer’s perspective – what they know and need |
| Recognises that communication with their employer* is key to a successful RTW outcome | Modelling *(Social Cognitive Theory)* | Providing an appropriate model | Identification with the model | Information in *RTW workbook, website* and *members of HOT* consistent in expressing their belief in communication with employer |
| Recognises that RTW is an ongoing process that needs to monitored | Elaboration *(Elaboration Likelihood Model)* | Stimulating the learner to add information that is processed | Messages that are personally relevant, easily understandable | *RTWC and HOT* (e.g. preassessment education) encourage discussion of RTW monitoring |
