# Supplementary material for: Using intervention mapping to develop an occupational advice intervention to aid return to work following hip and knee replacement in the United Kingdom
Source: BMC Health Serv Res. 2020 Jun 9;20:523. doi: 10.1186/s12913-020-05375-3 (PMC7285551; doi:10.1186/s12913-020-05375-3)
Supplement: Supplementary file 7 — Additional file 7. Parameters, methods and practical applications for staff determinants [file 12913_2020_5375_MOESM7_ESM.docx]

**Additional file 7. Parameters, methods and practical applications for Hospital Orthopaedic Team staff determinants.**

| KNOWLEDGE AND AWARENESS | | | |
| --- | --- | --- | --- |
| Methods | Definitions | Parameters | Applications |
| Discussion *(Elaboration Likelihood Model)* | Stimulating the learner to add meaning to the information that is processed | Listening to the learner to ensure that the correct schemas are activated | OPAL staff training prior to implementation. Ideally group, face-to-face, interactive |
| Elaboration *(Theories of Information Processing; Elaboration Likelihood Model)*  Coherence and imagery *(Theories of Information Processing)* | Stimulating the learner to add meaning to the information that is processed | Messages personally relevant, easily understandable | Each member of HOT has own OPAL study pack containing this information:  Study pack uses chunking, advance organisers and imagery methods to aid learning. I.e. sections of text have logical order and clearly related to each other using graphical representations  Each work area has study pack available  Computer-based version of training  Study website  Study newsletters |
| Individualisation/ tailoring *(Transtheoretical Model)* | Matching to participant characteristics, opportunities for personal/paced learning | Tailoring to participant, personal communication responds to learner’s need, relevance | Staff training tailored to specific profession/role/need  One-to-one training/support from OPAL team as required |
| Modelling *(Social Cognitive Theory; Theories of Learning)* | Providing an appropriate model | Identification with model  Coping v mastery model | Coping models of staff ‘tasks’ used in training/study packs |
| Consciousness raising *(Health Belief Model)*  Framing *(Protection Motivation Theory)* | Information about causes, and consequences of behaviour  Emphasise pros and cons of behaviour | Raising awareness should be quickly followed by increase in self-efficacy  Gain-frames more ready accepted | Staff training – consequences of providing RTW adivce/support |
| Providing cues *(Theories of Information Processing)* | Assuring same cues are present at time of learning and time of retrieval | Work best when people select and provide own cues | Staff at each research study site to suggest cues to action  e.g. Posters on ward/in clinic with photos of RTWC, OPAL champions and their contact details  e.g. OPAL study posters and pens |

| SKILLS AND SELF-EFFICACY | | | |
| --- | --- | --- | --- |
| Methods | Definitions | Parameters | Applications |
| Verbal persuasion *(Social Cognitive Theory)* | Use messages that suggest the participants possess certain capabilities | Credible source | Research team explain through training that they believe the HOT can do this; that OPAL study informed by stakeholders and evidence |
| Facilitation *(Social Cognitive Theory)* | Creating an environment that makes the action easier or reduces barriers | Required real changes in the environment | Staff training at optimal times/places/methods  e.g. Posters on ward/in clinic with photos of RTWC, OPAL champions and their contact details  e.g. Researchers and clinic team at each site establish easy/default methods of identifying RTW patients  e.g. Templates to facilitate completion of study documentation  e.g. ready supplies of study checklists, paperwork, pens  e.g. allowing sufficient time for staff performance objectives to be met |
| Information about others’ approval  Social Comparison *(Theory of Planned Behaviour; Social Comparison Theory)* | Providing information about whether others will approve or disapprove of any proposed behaviour change  Observation of other non-experts to evaluate one’s own opinions and abilities | Positive expectations available in environment  Upward comparison may help set better goals, downward may increase sense of self-efficacy | Staff training includes information on Phase 1 stakeholder interviews, and increasing focus on work and health  Comparison with other HOTs  Comparison with support for other health conditions  Study newsletters with updates from each site |
| Feedback  Reinforcement *(Theories of Learning, Goal Setting, Social Cognitive Theory)* | Giving information as to the extent of impact of performance  Lining behaviour to consequence that increases rate of behaviour | Feedback needs to be individual, specific and follow the behaviour in time  As above | Regular contact maintained with HOT from OPAL team  Study newsletters with updates from each site with positive feedback  Praise from OPAL team for staff engagement with OPAL study |
| Guided practice  Modelling *(Social Cognitive Theory)* | Prompting individuals to rehearse and repeat behaviour various times, discuss experience and provide feedback  Providing an appropriate model | Requires supervision by experienced person  Identification with model  Coping v mastery model | OPAL team members model/role play/provide examples of target behaviours then ask staff to do the same and give feedback emphasising what has been done well |
| Planning coping responses *(Attribution Theory; Theories of Self-Regulation)* | Prompting participants to list potential barriers and ways to overcome these | Identification of high-risk situations and practice of coping responses | OPAL team and HOT members discuss and problem-solve potential problems as part of training, e.g. patient avoids contact with RTWC, fails to bring RTW workbook |

| ATTITUDES, BELIEFS, EXPECTATIONS | | | |
| --- | --- | --- | --- |
| Methods | Definitions | Parameters | Applications |
| Self re-evaluation *(Trans-Theoretical Model)* | Encourage combining both cognitive and affective assessments of one’s self-image with and without an ‘unhealthy’ behaviour | Raising awareness must be quickly followed by increase in problem-solving ability and self-efficacy | Training encourages staff to focus on what they think and how they feel about being a HCP that supports patients in returning to work |
| Shifting perspective *(Theories of Stigma and Discrimination)* | Encouraging the perspective of another | Initiation from the perspective of the learner; needs imaginary competence | Encouraging staff to view a change in their clinical practice from the perspective of the patient returning to work and their employer, using examples from stakeholder interviews and cohort study in Phase 1 of OPAL study as part of training programme |
| Persuasive communication *(Diffusion of Innovations Theory)* | Guiding people towards the adoption of an idea or action by using arguments or other means | Messages need to be relevant and not too discrepant from the beliefs of the individual | Persuading staff that the delivery of work-focused advice and support at an early stage in the patients RTW process is possible |
| Belief selection *(Theory of Planned Behaviour)* | Using messages to strengthen positive beliefs, weaken negative beliefs and introduce new beliefs | Requires investigation of current beliefs of individual before intervening | Using evidence-based data on RTW to change staff beliefs about the proportion of working patients undergoing surgery |

| PERCEIVED NORMS | | | |
| --- | --- | --- | --- |
| Method | Definition | Parameters | Applications |
| Self re-evaluation *(Trans-Theoretical Model)*  Belief selection  Shifting perspective  Persuasive communication | See above examples | See above examples | Training to focus on encouraging staff to see it as good practice/in accordance with new thinking on work and health/feasible:  For HOTS to provide early support and advice to patients  For patients to RTW following surgery  That improved advice and support will facilitate timely and successful RTW  That these patients often receive little support elsewhere  That the number of working patients undergoing surgery is likely to increase |
