# Supplementary material for: Using intervention mapping to develop an occupational advice intervention to aid return to work following hip and knee replacement in the United Kingdom
Source: BMC Health Serv Res. 2020 Jun 9;20:523. doi: 10.1186/s12913-020-05375-3 (PMC7285551; doi:10.1186/s12913-020-05375-3)
Supplement: Supplementary file 8 — Additional file 8. Final patient performance objectives [file 12913_2020_5375_MOESM8_ESM.docx]

**Additional file 8.** **Final patient performance objectives**

| **Patient Performance Objectives (POs)** |
| --- |
| **PRE-SURGERY** |
| **PO.1** Patient completes occupational checklist prior to appointment with surgeon |
| **PO.2** Patient makes informed decision about surgery with respect to work |
| **PO.3** Patient acquaints self with key information about recovery and RTW provided in the RTW workbook |
| **PO.4** Patient brings RTW workbook to each hospital appointment including hospital inpatient stay (and discusses with Hospital Orthopaedic Team) |
| **PO.5** Patient completes sections of RTW workbook that will help them understand the demands of their work and set an approximate RTW date with employer* as required |
| **PO.6** Patient uses information resources provided in workbook to identify and prioritise potential barriers and solutions to a safe and appropriate RTW, and to develop a RTW plan with employer* as required |
| **PO.7** Patient discusses information within RTW workbook with RTW co-ordinator (at hospital or by phone) to help them further develop their RTW plan. This will include a minimum of 1 contact. The number and duration of further contacts will be governed by patient need based on progress and perceived level of ‘risk’ of prolonged sickness absence |
| **PO.8** Patient provides employer* with written information provided by the Hospital Orthopaedic Tem about their planned surgery and recovery/RTW advice |
| **POST SURGERY** |
| **PO.9** Patient meets with their employer* to discuss their recovery and RTW plan |
| **PO.10** Patient communicates with employer* regarding surgical outcome and progress/recovery |
| **PO.11** Patient revises RTW plan following surgery as necessary with their employer* and hospital staff |
| **PO.12** Patient engages with RTWC via RTW helpline/answering service if having problems related to RTW for up to 16 weeks post- surgery |
| **PO.13** Patient adheres to postoperative rehabilitation plan and advice |

*Not all patients will have an employer: Self-employed - POs referring to employer*s do not apply, although patient encouraged to undertake these objectives with colleagues/customers where appropriate. Carer - POs referring to employer*s do not apply, although patient encouraged to undertake these objectives with other stakeholders (e.g. recipient of care, co-carers) if appropriate. Volunteer - ‘Employer*’ may include manager/supervisor of voluntary work
