# Supplementary material for: Using intervention mapping to develop an occupational advice intervention to aid return to work following hip and knee replacement in the United Kingdom
Source: BMC Health Serv Res. 2020 Jun 9;20:523. doi: 10.1186/s12913-020-05375-3 (PMC7285551; doi:10.1186/s12913-020-05375-3)
Supplement: Supplementary file 9 — Additional file 9. Final performance objectives for members of the Hospital Orthopaedic Team [file 12913_2020_5375_MOESM9_ESM.docx]

**Additional file 9. Final Hospital Orthopaedic Team staff performance objectives**

| **PRE-SURGERY** |
| --- |
| **PO.1** The *Hospital Orthopaedic Team*:   - Identifies existing team members to act as *RTWC* and deputy - Identifies existing staff members to act as *OPAL Champions* for their team:   -ward  -inpatient therapy team  -outpatient clinic  -pre-assessment and education   - Develops a phone line / answerphone service for RTW patients to contact *RTWC* if they are having problems regarding RTW |
| **PO.2** *The outpatient clinic team* identifies RTW patients in clinic prior to consultation with surgical team |
| **PO.3** *The outpatient clinic team* requests RTW patients to complete occupational checklist prior to consultation with surgeon and explain its purpose to the patient, model completion if necessary and give positive feedback on completion  *The outpatient clinic team* gives completed occupational checklist to surgeon prior to patient’s appointment |
| **PO.4** *Surgeon* discusses pros and cons of surgery with patient including expected timescales of surgery and recovery – in relation to the patient’s usual work and refers to/responds positively to the patient’s occupational checklist to enable patient to make informed decision about surgery; supports patient autonomy  - Provides patient with personal risk feedback on potential RTW outcomes  -Explores patient’s questions and concerns  -Informs listed patients that they will be given a RTW workbook to read and why, complete where possible, bring to each subsequent appointment, presenting positive message  -Informs listed patients that they will receive an Employer* workbook and why, that the patient will be contacted by a RTWC at least 4 weeks prior to surgery and why. Names them.  -Explains that RTW plan may need to be revised and that RTWC will help with this  -Summarises and records patients RTW status/outcome in all clinic notes and following each appointment  -Communicates with GP at point patient is discharged from orthopaedic surgical care outlining current RTW status and progress and on-going therapy received |
| **PO.5** *The outpatient clinic team* provides all RTW patients listed for surgery with written RTW workbook and gain contact details for RTWC to contact patient as completed in occupational checklist  -Outpatient clinic staff inform/encourage patient to bring RTW workbook to each hospital appointment, and draw attention to this instruction in the workbook  -Discuss potential reasons why this might not happen, and formulate solutions with patient  -Recommend patients read workbook and complete as much as they can (show relevant sections); present workbook positively and refer to coping model examples  -Recommend patient asks employer* to assist patient in completion if wishes and suggests who this might include, and discuss possible difficulties and solutions re communicating with employer-Outpatient clinic staff explain to patient that the RTWC will contact them at least 4 weeks prior to surgery about their RTW plan |
| **PO.6** *The outpatient clinic team* provides all RTW patients listed for surgery with ‘Employer RTW workbook’ to share with their employer/colleagues*  - Outpatient clinic staff inform/encourage patient that giving the Employer RTW workbook to employer/ colleagues will help them understand surgery and prepare for patient’s RTW  -Suggests that patient might wish to meet with their employer to discuss RTW and who this might include  -Outpatient clinic staff suggest individuals in the workplace who might best receive the Employer TRW workbook |
| **PO.7** *The outpatient clinic team* collects patient’s completed occupational checklist from surgeon and forwards to RTWC |
| **PO.8** *The pre-operative assessment and education teams* routinely include the topic of RTW in their clinics with examples of work demands, barriers and facilitators to RTW, RTW plans, importance of adhering to postop rehab plan/pacing up activities  *-The pre-operative assessment and education teams* ask if patients have brought their RTW workbook to appointment, praise patients, refer positively to content and use of the workbooks, and promote engagement with the RTWC |
| **PO.9** *RTWC* contacts all RTW patients (phone/meet ups) at least 4 weeks prior to surgery to review:   - information provided in the occupational checklist - information in the RTW workbook including - Current job demands - Provisional RTW date - Potential barriers and solutions to safe and appropriate RTW - The patient’s provisional RTW plan   -All patients receive at least 1 contact with the RTW co-ordinator. This may be integrated within the pre-assessment / pre-admission process or done by phone. The number and duration of additional contacts will be governed by patient need based on progress and perceived level of ‘risk’  -Refers positively to RTW workbook during discussions with patient:   - Praises patient for bringing workbook to appointments - Reminds patient to bring workbook on admission - Refers to other patient examples /models of job demands/RTW plans etc   -Encourages discussion about/coaches patient regarding communication with patients employer  -Refers on/signposts where appropriate  -Sets goals/steps with patient  -Discusses the possibility of needing to revise RTW plan following surgery  -Documents all consultations in RTWC workbook |
| **PO.10** *RTWC* highlights RTW patients to teams managing *pre-operative education and assessment* and records this action in RTWC workbook |
| **PO.11** *RTWC* highlights RTW patients to *the ward teams* when admitted for surgery and records this action in the RTWC workbook |
| **PO.12** *The ward team (nurse and doctor)* check RTW patients have brought workbook into hospital and if not determine the reason for this. Give praise if workbook brought in. Refer positively to RTW workbook. |
| **POST-SURGERY** |
| **PO.13** *Ward therapists* ask RTW patients if they have brought workbook into hospital, and if not determine the reason for this. Give praise if workbook brought in.  Refer positively to RTW workbook, enter notes as appropriate  -Liaise with RTWC to update them on the patient’s postop recovery prior to discharge |
| **PO.14** *The RTWC* liaises with *inpatient teams* post-operatively to determine whether there are any issues with early recovery that may impact on the RTW plan  -The *RTWC* revises RTW plan with patient as required and ensures plan is documented in patients RTW workbook  -The *RTWC* supports post-operative rehab plans and problem-solves potential barriers to adherence with patient |
| **PO.15** *The ward team (nurse/doctor)* summarises patient’s expected RTW outcome and RTW plan in ward electronic discharge letter. A copy/copies will be given to the patient to share with employer, therapists etc.  *-The ward team (nurse/doctor)* praise/refer to the RTW workbook and remind the patient to use the RTW helpline following discharge if they are having problems  *-The ward team (nurse/doctor/therapist)* highlight the importance of adhering to the post op rehab plan |
| **PO.16** *The specialist ward nurse/doctor* asks each patient whether they require a fit note on discharge and completes the fit note in accordance with best practice guidelines and the hospital contract, and with reference to the patient’s RTW plan in their workbook |
| **PO.17** *The RTWC* checks the RTW helpline 3 x wk, and triages, advises (e.g. phone call) or refers back to therapy services (based on local service structure and availability) based on individual need. |
| **PO.18** *Surgeon, HOT and outpatient therapy teams* summarise and record patient’s RTW status / outcome in all outpatient clinic notes and following each appointment |
| **PO.19** *Surgeon and HOT* communicate with GP at point patient is discharged from orthopaedic surgical care, outlining current RTW status and progress and on-going therapy received and encourage engagement with RTWC until 16 weeks post-surgery (8 weeks for feasibility study) |
| **PO.20** *RTWC* continues to provide a point of access to RTW advice for patients following discharge from orthopaedic surgical care until 16 weeks post-surgery (8 weeks for feasibility study)  -Records any changes to patient’s RTW progress/status/outcome in RTWC workbook |

*Not all patients will have an employer: Self-employed - POs referring to employer*s do not apply, although patient encouraged to undertake these objectives with colleagues/customers where appropriate. Carer - POs referring to employer*s do not apply, although patient encouraged to undertake these objectives with other stakeholders (e.g. recipient of care, co-carers) if appropriate. Volunteer - ‘Employer*’ may include manager/supervisor of voluntary work
