# Supplementary material for: Using intervention mapping to develop an occupational advice intervention to aid return to work following hip and knee replacement in the United Kingdom
Source: BMC Health Serv Res. 2020 Jun 9;20:523. doi: 10.1186/s12913-020-05375-3 (PMC7285551; doi:10.1186/s12913-020-05375-3)
Supplement: Supplementary file 10 — Additional file 10. Final matrices of change and determinants for each patient performance objective [file 12913_2020_5375_MOESM10_ESM.docx]

**Additional file 10. Matrices of change for patients.**

RTW = return to work RTWC = return to work co-ordinator (a designated member of the hospital team) HOT = Hospital Orthopaedic Team

Behaviour to be targeted: Patient makes successful return to work following surgery

| **Performance Objective** | **Knowledge & Awareness** | **Skills & Self-efficacy** | **Attitudes/Beliefs/**  **Emotions** | **Outcome expectations** | **Perceived norms** |
| --- | --- | --- | --- | --- | --- |
| **PRE-SURGERY** |  |  |  |  |  |
| PO.1 Patient completes occupational checklist prior to appointment with surgeon | Explains that completing the occupational checklist aims to inform the surgeon about their work activities and demands | Expresses confidence in completing the occupational checklist | States that completing an occupational checklist will help to inform the surgeon about their work activities and demands and facilitate an informed decision about surgery | States that completing an occupational checklist  will facilitate an informed decision about surgery and positive RTW outcome | Recognises that nowadays patients are being encouraged to take an active part in their care  Recognises that RTW is now considered a health outcome |
| PO.2 Patient makes informed decision about surgery with respect to work | Appraises the general risks/benefits of surgery and RTW rates  Appraises the likely impact of surgery on their ability to do their own job  States that they have received sufficient information about surgery | Expresses confidence in ability to make informed decision about surgery  Demonstrates ability to process information about surgical procedure and make informed choice | Expresses willingness to take responsibility for surgical decision  Demonstrates appropriate emotional response with regard to their decision | Describes a realistic expectation of RTW outcome following surgery | Perceives that it is usual for patients to make an informed decision about surgery with respect to their work  Recognises that nowadays patients are being encouraged to take an active part in their care  Recognises that RTW is now considered a health outcome |
| PO.3 Patient acquaints self with key information about recovery and RTW provided in the RTW workbook | Describes the key advice and information concerning recovery and RTW e.g.  how work modifications (hours and duties) can facilitate RTW  the risks of extended sickness absence  the risks of RTW too quickly | Expresses confidence in their ability to acquaint themselves with key information about recovery and RTW provided in the RTW workbook | States they have a responsibility to acquaint themselves with key information about recovery and RTW provided in the RTW workbook  Expresses willingness to take this responsibility to acquaint themselves with key information? | States that having a good understanding about recovery and RTW is likely to lead to a positive RTW outcome | Recognises that patients undergoing surgery acquaint themselves with key information about recovery and RTW provided by the hospital orthopaedic team |
| PO.4 Patient brings RTW workbook to each hospital appointment including hospital inpatient stay (and discusses with HOT) | Describes that the reason for bringing the RTW workbook to each appointment is to encourage patients and hospital staff to focus on RTW at each appointment | Expresses confidence in their ability to bring the RTW workbook to each hospital appointment | States that it will help their recovery/RTW to bring the RTW workbook to each hospital appointment  Expresses willingness to bring RTW workbook to each hospital appointment | Expects that bringing the RTW workbook to each hospital appointment is likely to facilitate a positive RTW outcome | Recognises that nowadays patients are being encouraged to take an active part in their care |
| PO.5 Patient completes sections of RTW workbook that will help them understand the demands of their work and set an approximate RTW date  With employer* as required | Explains that completing the workbook helps them understand the demands of their work and set an approximate RTW date  Describes how to complete a RTW workbook and set an approximate RTW date, and how to do this with their  employer* if required | Expresses confidence in their ability to complete the sections of the RTW workbook that will help them understand the demands of their work and set an approximate RTW date  Expresses confidence in their ability to do this with their employer* if required | Expresses willingness to complete the sections of RTW workbook that will help them understand the demands of their work and set an approximate RTW date  Expresses willingness to do this with their employer* if required | Expects that completing the sections of the RTW workbook (with their employer if required) that will help them understand the demands of their work and set an approximate RTW date is likely to lead to a positive RTW outcome | Recognises that RTW is now considered a health outcome  Recognises that nowadays patients are being encouraged to take an active part in their care  Recognises that employers* are key stakeholders in RTW and involving them at an early stage can facilitate RTW |
| PO.6 Patient uses information resources provided in workbook to identify and prioritise potential barriers and solutions to a safe and appropriate RTW, and to develop a RTW plan  With employer* as required | Lists the potential barriers and solutions to their own RTW and develops a RTW plan, with employer as required.  Explains how to identify and prioritise potential barriers and solutions to a safe and appropriate RTW and develop a RTW plan, with employer as required | Expresses confidence in identifying barriers/facilitators to their own safe and appropriate RTW, and to develop a RTW plan, with employer as required | States that identifying barriers/facilitators and the development of their RTW plan, with employer as required, will aid their own safe and appropriate RTW | Expects that identifying and prioritising potential barriers and facilitators to RTW, and developing a RTW plan, with employer as required, will lead to a safe and appropriate RTW | Recognises that nowadays patients are being encouraged to take an active part in their care  Recognises that employers* are key stakeholders in RTW and involving them at an early stage can facilitate RTW |
| PO.7 Patient discusses information within RTW workbook with RTW co-ordinator (at hospital or by phone) to help them further develop their RTW plan. This will include a minimum of 1 contact. The number and duration of further contacts will be governed by patient need based on progress and perceived level of ‘risk’ of prolonged sickness absence | Describes the process of engaging with their RTWC to further develop a RTW plan:   - How - When - Where | Expresses confidence in engaging with the RTWC to help them further develop their a RTW plan  Demonstrates how to negotiate a RTW plan with their employer* | States that engaging with the RTWC to help them further their RTW plan will aid their RTW  Expresses acceptance that a RTW plan will aid their RTW | Expects that engaging with the RTWC to help them further develop their RTW plan will lead to a positive RTW outcome | Recognises that the ideal RTW process relies on coordination and joint planning between healthcare, the patient and their employer |
| PO.8 Patient provides employer* with written information provided by the HOT about their planned surgery and recovery/RTW advice | Describes the information that they can provide to their employer*/workplace, and who should receive it | Expresses confidence in their ability to provide this information to their employer*/workplace | States that providing their employer* with written information provided by the HOT about their planned surgery and recovery/RTW advice will facilitate their RTW. | Employer* is informed about the surgical process and RTW | Recognises that employers do not necessarily know about this type of surgery and how best to facilitate RTW  Recognises that employers* are key stakeholders in RTW and involving them at an early stage can facilitate RTW |
| **POST SURGERY** |  |  |  |  |  |
| PO.9 Patient meets with their employer* to discuss their recovery and RTW plan | Appraises the likely impact of surgery on their RTW, prior to their operation  Describes how to discuss their RTW with their employer* | Expresses their ability to discuss their recovery and RTW plan with their  employer*/workplace  Expresses confidence in their ability to discuss their recovery and RTW plan with their  employer*/workplace | Expresses willingness to discuss their recovery and RTW plan with their employer* | Employer* is informed about patient’s recovery and RTW plan | Recognises that nowadays patients are being encouraged to take an active part in their care  Recognises that employers* are key stakeholders in RTW and involving them at an early stage can facilitate RTW |
| PO.10 Patient communicates with employer* regarding surgical outcome and progress/recovery | Appraises the likely impact of surgery on their RTW, post-surgery | Expresses confidence in their ability to communicate with their employer* regarding surgical outcomes and recovery (could be by phone, in writing, in person) | States their willingness to communicate their surgical outcome and progress with their employer* | Expects that communicating with their employer* regarding surgical outcome and progress will lead to a positive RTW outcome | Recognises that communication with their employer* is key to a successful RTW outcome |
| PO.11 Patient revises RTW plan following surgery as necessary with their employer* and hospital staff | Explains why a RTW plan may need to be revised following surgery  Describes how they will revise their RTW plan if necessary with their employer* and hospital staff | Expresses confidence in negotiating a revised RTW plan with their employer* and hospital staff | States their willingness to revise their RTW plan following surgery | Expects that revising the RTW plan following surgery will provide a more positive RTW experience | Recognises that nowadays patients are being encouraged to take an active part in their care  Recognises that the ideal RTW process relies on coordination and joint planning between healthcare, the patient and their employer  Recognise that RTW is an ongoing process that needs to monitored |
| PO.12 Patient engages with RTWC via RTW helpline/answering service if having problems related to RTW for up to 16 weeks post- surgery | Recalls the process of engaging with the RTWC:   - Who to contact - How to contact them - When to contact them - What action is to be expected and when | Expresses confidence in their ability to engage with the RTWC if they are having problems post-discharge | States that engaging with the RTWC via the RTW helpline/answering service will potentially alleviate any RTW problems  Expresses willingness to engage with this service if problems relating to RTW emerge | Expects that engaging with the RTWC via RTW helpline/answering service if having problems related to RTW will help the patient to overcome the problem | Recognises that it is considered normal for patients to ask clinicians for help regarding problems at work, even after discharge from the service |
| PO.13 Patient adheres to postoperative rehabilitation plan and advice | Describes their postoperative rehabilitation plan:   - What - When - Where - Who with   Describes risks of not adhering to rehabilitation plan | Expresses ability to attend/travel to postoperative rehabilitation sessions if required  Expresses confidence about adhering to postoperative rehabilitation plan | States that adhering to their postoperative rehabilitation plan is important for their recovery/RTW  Expresses willingness to adhere to postoperative rehabilitation plan and advice | Expects that adhering to their postoperative rehabilitation plan will have a positive impact on RTW | Recognises that other patients undergoing surgery take an active part in postop rehabilitation  Recognises that nowadays patients are being encouraged to take an active part in their care |
