# Supplementary material for: Using intervention mapping to develop an occupational advice intervention to aid return to work following hip and knee replacement in the United Kingdom
Source: BMC Health Serv Res. 2020 Jun 9;20:523. doi: 10.1186/s12913-020-05375-3 (PMC7285551; doi:10.1186/s12913-020-05375-3)
Supplement: Supplementary file 11 — Additional file 11. Final matrices of change and determinants for each Hospital Orthopaedic Team performance objective [file 12913_2020_5375_MOESM11_ESM.docx]

**Additional file 11. Matrices of change for Hospital Orthopaedic Team staff**

HOT = Hospital Orthopaedic Team

OPALC = OPAL Champion. Each within the HOT to have an identified OPALC who is responsible for ensuring that a member/members of their team meet the performance objectives

RTW = return to work

RTWC = return to work co-ordinator (an existing member of the HOT team trained up for this role, e.g. nurse, physio, occupational therapist)

| **Performance Objective** | **Knowledge & Awareness** | **Skills & Self-efficacy** | **Attitudes/Beliefs/Expectations** | **Perceived norms** |
| --- | --- | --- | --- | --- |
| PRE-SURGERY |  |  |  |  |
| PO.1 The *HOT*:   - Identifies existing team members to act as *RTWC* and deputy - Identifies existing staff members to act as *OPALCs* for their team:   -ward  -inpatient therapy team  -outpatient clinic  -pre-assessment and education   - Develops a phone line / answerphone service for RTW patients to contact *RTWC* if they are having problems regarding RTW | Members of HOT describe role and responsibility of the RTWC and OPALCs  Members of HOT state identity of the RTWC, their deputy, and OPALCs  Members of HOT describe how to contact the RTWC, their deputy, and OPALCs  Members of HOT describe how patients will use the phone line / answerphone service to contact the RTWC | Members of the HOT are confident that they are able to   - Identify existing team members to act as RTWC and Deputy - Identify existing staff members to act as *OPALCs* for their team:   -ward  -inpatient therapy team  -outpatient clinic  -pre-assessment and education   - Develop a phone line / answerphone service for RTW patients to contact *RTWC* if they are having problems regarding RTW | Members of the HOT state that the following actions will facilitate patients in RTW:   - Identifying existing team members to act as RTWC and Deputy - Identifying existing staff members to act as *OPALCs* for their team:   -ward  -inpatient therapy team  -outpatient clinic  -pre-assessment and education   - Developing a phone line / answerphone service for RTW patients to contact *RTWC* if they are having problems regarding RTW | Members of the HOT recognise that the NHS now sees RTW as a measure of health and recovery from surgery  Members of HOT recognise that patients undergoing THR and TKR are increasingly likely to RTW following surgery  Members of the HOT recognise that HOTs have a role in supporting patients undergoing THR/TKR in RTW following surgery |
| PO.2 *The outpatient clinic team* identifies RTW patients in clinic prior to consultation with surgical team | Members of the outpatient clinic team describe the process of identifying RTW patients:   - how - when - where | Members of the outpatient clinic team express confidence in their ability to identify RTW patients in clinic | Members of the outpatient clinic team state that identifying RTW patients in clinic will help the surgeon / patient make an informed decision about surgery with regard to RTW | Members of the outpatient clinic team recognise that identifying RTW patients in clinic prior to appointment with surgeon is good practice |
| PO.3 *The outpatient clinic team* requests RTW patients to complete occupational checklist prior to consultation with surgeo nand explain its purpose to the patient, model completion if necessary and give positive feedback on completion  *The outpatient clinic team* gives completed occupational checklist to surgeon prior to patient’s appointment | Members of the outpatient clinic team describe the process of asking RTW patients to complete an occupational checklist and giving it to the surgeon:   - how - when - where   Members of the outpatient clinic team describe the process of modelling completion of the occupational checklist and giving positive feedback on its completion | Members of the outpatient clinic team express confidence in their ability to ask RTW patients to complete an occupational checklist in clinic  and giving it to the surgeon prior to patient’s appointment  Members of the outpatient clinic team express confidence in modelling completion of the occupational checklist and giving positive feedback on its completion | Members of the outpatient clinic team state that asking RTW patients to complete an occupational checklist in clinic will help the surgeon and patient make a more informed decision about surgery with regard to RTW  Members of the outpatient clinic team state that modelling completion of the occupational checklist and giving positive feedback on its completion will help the patient to complete the checklist accurately and help the patient and surgeon make a more informed decision about surgery with regard to RTW | Members of the outpatient clinic team recognise that preparing the patient and surgeon to discuss the patient’s RTW patients is good practice  Members of the outpatient clinic team state that modelling completion of the occupational checklist and giving positive feedback on its completion is in accordance with good practice |
| PO.4 *Surgeon* discusses pros and cons of surgery with patient including expected timescales of surgery and recovery – in relation to the patient’s usual work and refers to/responds positively to the patient’s occupational checklist to enable patient to make informed decision about surgery; supports patient autonomy  Provides patient with personal risk feedback on potential RTW outcomes  Explores patients questions and concerns  Informs listed patients that they will be given a RTW workbook to read and why, complete where possible, bring to each subsequent appointment, presenting positive message  Informs listed patients that they will receive an Employer workbook and why, that the patient will be contacted by a RTWC at least 4 weeks prior to surgery and why. Names them.  Explains that RTW plan may need to be revised and that RTWC will help with this  Summarises and records patients RTW status/outcome in all clinic notes and following each appointment  Communicates with GP at point patient is discharged from orthopaedic surgical care outlining current RTW status and progress and on-going therapy received | Surgeon describes current evidence regarding pros and cons of surgery in relation to work including expected timescales of surgery and recovery  Surgeon describes the process by which they use occupational checklist  Surgeon describes process of providing patient with personal risk feedback on potential RTW outcomes  Surgeon describes process of enabling patient to make informed decision about surgery; supporting patient autonomy  Surgeon describes the process of:  -Exploring patients questions and concerns  -Informing listed patients that they will be given a RTW workbook to read and why, complete where possible, bring to each subsequent appointment, presenting positive message  -Informing listed patients that they will receive an Employer workbook and why, that the patient will be contacted by a RTWC at least 4 weeks prior to surgery and why. Names them.  -Explaining that RTW plan may need to be revised and that RTWC will help with this  -Summarising and recording patients RTW status/outcome in all clinic notes and following each appointment  -Communicating with GP at point patient is discharged from orthopaedic surgical care outlining current RTW status and progress and on-going therapy received | Surgeon expresses confidence in discussing/answering patient’s questions about RTW and their decision to have surgery in relation to their work  Surgeon expresses confidence in using the patient’s occupational checklist as a basis for their discussion with patient about surgery  Surgeon expresses confidence in providing patient with personal risk feedback on potential RTW outcomes  Surgeon expresses confidence in enabling patient to make informed decision about surgery; supporting patient autonomy  Surgeon expresses confidence in:  -Exploring patients questions and concerns  -Informing listed patients that they will be given a RTW workbook to read and why, complete where possible, bring to each subsequent appointment, presenting positive message  -Informing listed patients that they will receive an Employer workbook and why, that the patient will be contacted by a RTWC at least 4 weeks prior to surgery and why. Names them.  -Explaining that RTW plan may need to be revised and that RTWC will help with this  -Summarising and recording patients RTW status/outcome in all clinic notes and following each appointment  -Communicating with GP at point patient is discharged from orthopaedic surgical care outlining current RTW status and progress and on-going therapy received | Surgeon states that surgeons should encourage patients to take an active role in the decision about surgery in relation to RTW  Surgeon states that using the patient’s occupational checklist as a basis for their discussion about surgery will facilitate their discussion about surgery  Surgeon states that providing patient with personal risk feedback on potential RTW outcomes and enabling patient to make informed decision about surgery – supporting patient autonomy - will facilitate their RTW  Surgeon states that the patient's RTW will be facilitated by:  -Exploring patients questions and concerns  -Informing listed patients that they will be given a RTW workbook to read and why, complete where possible, bring to each subsequent appointment, presenting positive message  -Informing listed patients that they will receive an Employer workbook and why, that the patient will be contacted by a RTWC at least 4 weeks prior to surgery and why. Names them.  -Explaining that RTW plan may need to be revised and that RTWC will help with this  -Summarising and recording patients RTW status/outcome in all clinic notes and following each appointment  -Communicating with GP at point patient is discharged from orthopaedic surgical care outlining current RTW status and progress and on-going therapy received | Surgeons recognise that discussing the pros and cons of surgery with patient including expected timescales of surgery and recovery – in relation to the patient’s usual work - is good practice  Surgeon states that using the patient’s occupational checklist as a basis for their discussion about surgery is good practice  Surgeon states that providing patient with personal risk feedback on potential RTW outcomes and enabling patient to make informed decision about surgery – supporting patient autonomy -is good practice  Surgeon recognises that it is good practice to:  -Exploring patients questions and concerns  -Inform listed patients that they will be given a RTW workbook to read and why, complete where possible, bring to each subsequent appointment, presenting positive message  -Inform listed patients that they will receive an Employer workbook and why, that the patient will be contacted by a RTWC at least 4 weeks prior to surgery and why. Names them.  -Explain that RTW plan may need to be revised and that RTWC will help with this  -Summarise and record patients RTW status/outcome in all clinic notes and following each appointment  -Communicate with GP at point patient is discharged from orthopaedic surgical care outlining current RTW status and progress and on-going therapy received |
| PO.5 *The outpatient clinic team* provides all RTW patients listed for surgery with written RTW workbook and gain contact details for RTWC to contact patient as completed in occupational checklist  Outpatient clinic staff inform/encourage patient to bring RTW workbook to each hospital appointment, and draw attention to this instruction in the workbook  Discuss potential reasons why this might not happen, and formulate solutions with patient  Recommend patients read workbook and complete as much as they can (show relevant sections); present workbook positively and refer to coping model examples  Recommend patient asks employer to assist patient in completion if wishes and suggests who this might include, and discuss possible difficulties and solutions re communicating with employer  Outpatient clinic staff explain to patient that the RTWC will contact them at least 4 weeks prior to surgery about their RTW plan | Members of the outpatient clinic team describe the process of giving patients a RTW workbook and gaining contact details for RTWC to contact patient:   - how - when - where   Outpatient clinic staff describe the process of:  - informing/encouraging patient to bring RTW workbook to each hospital appointment, and drawing attention to this instruction in the workbook  - discussing potential reasons why this might not happen, and formulating solutions with patient  Outpatient clinic staff describe the process of recommending that patients read workbook and complete as much as they can (show relevant sections) ; presenting workbook positively and referring to coping model examples  Outpatient clinic staff describe the process of recommending that patients asks employer to assist patient in completion if wishes and suggests who this might include, and discuss possible difficulties and solutions re communicating with employer  Outpatient clinic staff describe the process of explaining to patient that the RTWC will contact them about their RTW plan | Members of the outpatient clinic team express confidence in their ability to give patients a RTW workbook and gain contact details for RTWC to contact patient  Outpatient clinic staff express confidence in their ability to:  - inform/encourage patient to bring RTW workbook to each hospital appointment, and to draw attention to this instruction in the workbook  - discuss potential reasons why this might not happen, and formulating solutions with patient  Outpatient clinic staff express confidence in their ability to of recommend to patients that they read workbook and complete as much as they can (show relevant sections); presenting the workbook positively and referring to coping model examples  Outpatient clinic staff express confidence in recommending that patients asks employer to assist patient in completion if wishes and suggests who this might include, and discuss possible difficulties and solutions re communicating with employer  Outpatient clinic staff express confidence in their ability to explain to patient that the RTWC will contact them about their RTW plan | Members of the outpatient clinic team state that giving patients a RTW workbook and RTWC contact phone/email will facilitate the patient’s RTW  Outpatient clinic staff state that:  - informing/encouraging patient to bring RTW workbook to each hospital appointment, and drawing attention to this instruction in the workbook and discussing potential reasons why this might not happen/formulating solutions will facilitate their RTW  Outpatient clinic state that recommending to patients that they read workbook and complete as much as they can (show relevant sections); presenting the workbook positively and referring to coping model examples will facilitate the patient’s RTW    Outpatient clinic staff state that recommending that patients asks employer to assist patient in completion if wishes and suggesting who this might include, and discussing possible difficulties and solutions re communicating with employer  will facilitate their RTW  Outpatient clinic staff state that explaining to patient that the RTWC will contact them about their RTW plan will facilitate RTW | Members of the outpatient clinic team recognise that it is good practice to give patients RTW information and support at an early stage  Outpatient clinic staff recognise that informing/encouraging patient to bring RTW workbook to each hospital appointment, drawing attention to this instruction, and discussing potential reasons why this might not happen, and formulating solutions with the patient is good practice  Outpatient clinic recognise that recommending to patients that they read workbook and complete as much as they can (show relevant sections); presenting the workbook positively and referring to coping model examples is good practice  Outpatient clinic staff recognise that recommending that patients asks employer to assist patient in completion if wishes and suggesting who this might include, and discussing possible difficulties and solutions re communicating with employer is good practice  Outpatient clinic staff recognise that explaining to patient that the RTWC will contact them about their RTW plan is good practice |
| PO.6 *The outpatient clinic team* provides all RTW patients listed for surgery with ‘Employer RTW workbook’ to share with their employer/colleagues*  Outpatient clinic staff inform/encourage patient that giving the Employer RTW workbook to employer/ colleagues will help them understand surgery and prepare for patient’s RTW  Suggests that patient might wish to meet with their employer to discuss RTW and who this might include  Outpatient clinic staff suggest individuals in the workplace who might best receive the Employer TRW workbook | Members of the outpatient clinic team describe the process of giving patients the Employer RTW workbook to share with their employer/colleagues*:   - how - when - where   Outpatient clinic staff describe process of informing/encouraging patient that giving an Employer RTW workbook to their employer/ colleagues will help them understand surgery and prepare for patient’s RTW  Outpatient clinic staff describe the process of recommending that patients might wish to meet with their employer to discuss RTW and who this might include  Outpatient clinic staff describe process of suggesting individuals in the workplace who might best receive the Employer RTW workbook | Members of the outpatient clinic team express confidence in their ability to provide patients with Employer RTW workbook  Outpatient clinic staff express confidence in their ability to inform/encourage patient that giving the ‘Employer RTW workbook’ to employer/ colleagues will help them understand surgery and prepare for patient’s RTW  Outpatient clinic staff express confidence in recommending that patients might wish to meet with their employer to discuss RTW and who this might include  Outpatient clinic staff express confidence in their ability to suggest individuals in the workplace who might best receive the employer information | Members of the outpatient clinic team state that giving patients an Employer RTW workbook to share with their employer/colleagues will facilitate the patient’s RTW  Outpatient clinic staff state that informing/encouraging patient to give the ‘Employer RTW workbook’ to employer/ colleagues will help them understand surgery and prepare for patient’s RTW  Outpatient clinic staff state that recommending that patients might wish to meet with their employer to discuss RTW and who this might include will facilitate their RTW  Outpatient clinic staff state that suggesting individuals in the workplace who might best receive the employer information will facilitate the patient’s RTW | Members of the outpatient clinic team recognise that it is good practice to educate/inform patients’ employers/colleagues* about RTW information at an early stage  Outpatient clinic staff recognise that it is good practice to inform/encourage patient to give the ‘Employer RTW workbook’ to employer/ colleagues.  Outpatient clinic staff recognises that recommending that patients might wish to meet with their employer to discuss RTW and who this might include is good practice  Outpatient clinic staff recognise that it is good practice to suggest individuals in the workplace who might best receive the Employer RTW workbook |
| PO.7 *The outpatient clinic team* collects patient’s completed occupational checklist from surgeon and forwards to RTWC | Members of the outpatient clinic team describe the process of passing patients’ completed occupational checklists to RTWC   - how - when - where | Members of the outpatient clinic express confidence in their ability to pass patients’ completed occupational checklists to RTWC | Members of the outpatient clinic team state that passing patients’ completed occupational checklists to RTWC will help RTWC facilitate the patient’s RTW | Members of the outpatient clinic team recognise that it is good practice for HOTs to communicate patients occupational status to RTWC |
| PO.8 *The pre-operative assessment and education teams* routinely include the topic of RTW in their clinics with examples of work demands, barriers and facilitators to RTW, RTW plans, importance of adhering to postop rehab plan/pacing up activities  *The pre-operative assessment and education teams* ask if patients have brought their RTW workbook to appointment, praise patients, refer positively to content and use of the workbooks, and promote engagement with the RTWC | Members of the preop assessment and education teams describe how to routinely include the topic of RTW in their clinics with examples of work demands, barriers and facilitators to RTW, RTW plans, importance of adhering to postop rehab plan/pacing up activities  Members of the pre-operative assessment and education teams describe the process of asking if patients have brought their RTW workbook to appointment, praising patients and referring positively to content and use of the workbooks, and promoting engagement with the RTWC | Members of preop assessment and education team express confidence in routinely include the topic of RTW in their clinics with examples of work demands, barriers and facilitators to RTW, RTW plans, importance of adhering to postop rehab plan/pacing up activities  Members of the pre-operative assessment and education teams express confidence in asking if patients have brought their RTW workbook to appointment, praising patients and referring positively to content and use of the workbooks, and promoting engagement with the RTWC | Members of preop assessment and education team state that routinely including the topic of RTW in their clinics with examples of work demands, barriers and facilitators to RTW, RTW plans, importance of adhering to postop rehab plan/pacing up activities will facilitate the patient’s decision about surgery and their RTW  Members of the pre-operative assessment and education teams state that asking if patients have brought their RTW workbook to appointment, praising patients and referring positively to content and use of the workbooks, and promoting engagement with the RTWC will facilitate the patients RTW | Members of preop assessment and education team recognise that routinely including the topic of RTW in their clinics with examples of work demands, barriers and facilitators to RTW, RTW plans, importance of adhering to postop rehab plan/pacing up activities  is good practice  Members of the pre-operative assessment and education teams recognise that asking if patients have brought their RTW workbook to appointment, praising patients and referring positively to content and use of the workbooks, and promoting engagement with the RTWC is good practice |
| PO.9 *RTWC* contacts all RTW patients (phone/meet ups) at least 4 weeks prior to surgery to review:   - information provided in the occupational checklist - information in the RTW workbook including - Current job demands - Provisional RTW date - Potential barriers and solutions to safe and appropriate RTW - The patient’s provisional RTW plan   All patients receive at least 1 contact with the RTW co-ordinator. This may be integrated within the pre-assessment / pre-admission process or done by phone. The number and duration of additional contacts will be governed by patient need based on progress and perceived level of ‘risk’  Refers positively to RTW workbook during discussions with patient:   - Praises patient for bringing workbook to appointments - Reminds patient to bring workbook on admission - Refers to other patient examples /models of job demands/RTW plans etc   Encourages discussion about/coaches patient regarding communication with patients employer  Refers on/signposts where appropriate  Sets goals/steps with patient  Discusses the possibility of needing to revise RTW plan following surgery  Documents all consultations in RTWC workbook | The RTWC describes the process of how, when and where they will:   - Contact RTW patients - Review the patients occupational checklist - Review information in the RTW workbook including - Current job demands - Provisional RTW date - Potential barriers and solutions to safe and appropriate RTW - The patient’s provisional RTW plan - Encourage discussion about/coach patient regarding communication with patients employer - Discuss the possibility of needing to revise RTW plan following surgery - Determine the number of patient contacts - Refer positively to RTW workbook during discussions with patient: - Praise patient for bringing workbook to appointments - Remind patient to bring workbook on admission - Refer to other patient examples /models of job demands/RTW plans etc - Refer on/signpost where appropriate - Sets goals/steps with patient - Discuss the possibility of needing to revise RTW plan following surgery - Documenting all consultations in RTWC workbook | The RTWC expresses confidence in their ability to:   - Contact RTW patients - Review the patients occupational checklist - Review information in the RTW workbook including - Current job demands - Provisional RTW date - Potential barriers and solutions to safe and appropriate RTW - The patient’s provisional RTW plan - Encourage discussion about/coach patient regarding communication with patients employer - Discuss the possibility of needing to revise RTW plan following surgery - Determine the number of patient contacts - Refer positively to RTW workbook during discussions with patient: - Praise patient for bringing workbook to appointments - Remind patient to bring workbook on admission - Refer to other patient examples /models of job demands/RTW plans etc - Refer on/signpost where appropriate - Set goals/steps with patient - Discuss the possibility of needing to revise RTW plan following surgery - Documenting all consultations in RTWC workbook | The RTWC states that by providing targeted individual RTW support and advice through contacting patients prior to surgery will facilitate their RTW | The RTWC recognises that providing targeted individual RTW support and advice through an contacting patients prior to surgery is good practice |
| PO.10 *RTWC* highlights RTW patients to teams managing *pre-operative education and assessment* and records this action in RTWC workbook | The RTWC describes the process of highlighting RTW patients to the pre-operative education and assessment team and recording this action in RTWC workbook   - How - When - Where | The RTWC expresses confidence in their ability to highlight RTW patients to the pre-operative education and assessment team and recording this action in RTWC workbook | The RTWC states that highlighting RTW patients to the pre-operative education and assessment team and recording this action in RTWC workbook  will facilitate the patient’s decision about surgery and their RTW | The RTWC recognises that highlighting RTW patients to the pre-operative education and assessment team and recording this action in RTWC workbook is good practice |
| PO.11 *RTWC* highlights RTW patients to *the ward teams* when admitted for surgery and records this action in the RTWC workbook | The RTWC describes the process of highlighting RTW patients to the ward team and recording this action in RTWC workbook when patient admitted:   - How - When - Where | The RTWC expresses confidence in their ability to highlight RTW patients to the ward team and recording this action in RTWC workbook | The RTWC states that highlighting RTW patients to the ward team and recording this action in RTWC workbook  will facilitate the patient’s RTW | The RTWC states that highlighting RTW patients to the ward team and recording this action in RTWC workbook is good practice |
| PO.12 *The ward team (nurse and doctor)* check RTW patients have brought workbook into hospital and if not determine the reason for this. Give praise if workbook brought in.  Refer positively to RTW workbook. | *The ward team (nurse and doctor)* describe the process of checking that RTW patients have brought workbook into hospital, and if not, determining the reason for this; giving praise if workbook brought in; referring positively to RTW workbook. | *The ward team (nurse and doctor)* describe the process of checking that RTW patients have brought workbook into hospital, and if not, determining the reason for this. Give praise if workbook brought in.  Refer positively to RTW workbook. | *The ward team (nurse and doctor)* state that checking that RTW patients have brought workbook into hospital, and if not, determining the reason for this, giving praise if workbook brought in and referring positively to RTW workbook will facilitate the patient's RTW. | *The ward team (nurse and doctor)* recognise that checking that RTW patients have brought workbook into hospital, and if not, determining the reason for this, giving praise if workbook brought in and referring positively to RTW workbook is best practice. |
| **POST-SURGERY** |  |  |  |  |
| PO.13 *Ward therapists* ask RTW patients if they have brought workbook into hospital, and if not determine the reason for this. Give praise if workbook brought in.  Refer positively to RTW workbook, enter notes as appropriate  Liaise with RTWC to update them on the patient’s postop recovery prior to discharge | *Ward therapists* describe the process of:  - asking RTW patients if they have brought workbook into hospital, and if not determine the reason for this. Give praise if workbook brought in.  -Referring positively to RTW workbook, and entering in notes as appropriate  -Liaising with RTWC to update them on the patient’s postop recovery prior to discharge | *Ward therapists* express confidence in  - asking RTW patients if they have brought workbook into hospital, and if not determining the reason for this. Giving praise if workbook brought in.  -Referring positively to RTW workbook, and entering in notes as appropriate  -Liaising with RTWC to update them on the patient’s postop recovery prior to discharge | *Ward therapists* state that:  - asking RTW patients if they have brought workbook into hospital, and if not determine the reason for this and giving praise if workbook brought in.  -Referring positively to RTW workbook, and entering in notes as appropriate  -Liaising with RTWC to update them on the patient’s postop recovery prior to discharge  Will facilitate RTW. | *Ward therapists* recognise that it is good practice to:  - ask RTW patients if they have brought workbook into hospital, and if not determine the reason for this, and give praise if workbook brought in.  -refer positively to RTW workbook, and enter in notes as appropriate  -Liaise with RTWC to update them on the patient’s postop recovery prior to discharge |
| PO.14 *The RTWC* liaises with *inpatient teams* post-operatively to determine whether there are any issues with early recovery that may impact on the RTW plan  The *RTWC* revises RTW plan with patient as required and ensures plan is documented in patients RTW workbook  The *RTWC* supports post-operative rehab plans and problem-solves potential barriers to adherence with patient | The RTWC describes the process of liaising with inpatient teams post-operatively to determine whether there are any issues with early recovery that may impact on the RTW plan:   - How - When - Where   The *RTWC* describes the process of revising the RTW plan with patient as required and ensures plan is documented in patients RTW workbook  The *RTWC* describes the process of supporting post-operative rehab plans and problem-solving potential barriers to adherence with patient | The RTWC expresses confidence in their ability to liaise with the inpatient therapy team regarding patient’s post-op recovery  The *RTWC* expresses confidence in revising the RTW plan with patient as required and ensuring plan is documented in patients RTW workbook  The *RTWC* expresses confidence in supporting post-operative rehab plans and problem-solving potential barriers to adherence with patient | The RTWC states that liaising with the inpatient therapy team regarding patient’s post-op recovery will facilitate the patient’s RTW  The *RTWC* states that revising the RTW plan with patient as required and ensuring plan is documented in patients RTW workbook will facilitate the patient’s RTW  The *RTWC* states that supporting post-operative rehab plans and problem-solving potential barriers to adherence with patient will facilitate the patient’s RTW | The RTWC recognises that liaising with the inpatient therapy regarding patient’s post-op recovery is good practice  The *RTWC* states that revising the RTW plan with patient as required and ensuring plan is documented in patients RTW workbook is good practice  The *RTWC* states that supporting post-operative rehab plans and problem-solving potential barriers to adherence with patient  is good practice |
| PO.15 *The ward team (nurse/doctor)* summarises patient’s expected RTW outcome and RTW plan in ward electronic discharge letter. A copy/copies will be given to the patient to share with employer, therapists etc.  *The ward team (nurse/doctor)* praise/refer to the RTW workbook and remind the patient to use the RTW helpline following discharge if they are having problems  *The ward team (nurse/doctor/therapist)* highlight the importance of adhering to the post op rehab plan | The ward nurse and doctor describe how to  summarises the patient’s expected RTW outcome and RTW plan in ward electronic discharge letter  The ward nurse and doctor describe how a copy/copies will be given to the patient to share with employer, therapists  *The ward team (nurse/doctor)* describe the process of praising/referring to the RTW workbook and reminding the patient to use the RTW helpline following discharge if they are having problems  *The ward team (nurse/doctor/therapist)* describe the process of highlighting the importance of adhering to the post op rehab plan | The ward nurse and doctor express confidence in their ability to summarise the patient’s expected RTW outcome and RTW plan in ward electronic discharge letter  The ward nurse express confidence in their ability to give a copy/copies of the discharge letter to the patient to share with employer, therapists  *The ward team (nurse/doctor)* express confidence in praising/referring to the RTW workbook and reminding the patient to use the RTW helpline following discharge if they are having problems  *The ward team (nurse/doctor/therapist)* express confidence in highlighting the importance of adhering to the post op rehab plan | The ward nurse and doctor state that summarising the patient’s expected RTW outcome and plan in the ward electronic discharge letter will facilitate the patient’s RTW  The ward nurse and doctor state that giving the patient a copy/copies of the electronic discharge letter to share with their employer, therapists etc will facilitate the patient’s RTW  *The ward team (nurse/doctor)* state that praising/referring to the RTW workbook and reminding the patient to use the RTW helpline following discharge if they are having problems will facilitate their RTW  *The ward team (nurse/doctor/therapist)* state highlighting the importance of adhering to the post op rehab plan will facilitate their RTW | The ward nurse and doctor recognise that summarising the patient’s expected RTW outcome and plan in the ward electronic discharge letter is good practice  The ward nurse and doctor recognise that giving the patient a copy/copies of the electronic discharge letter to share with their employer, therapists etc is good practice  *The ward team (nurse/doctor)* recognise that praising/referring to the RTW workbook and reminding the patient to use the RTW helpline following discharge if they are having problems is good practice  *The ward team (nurse/doctor/therapist)* state highlighting the importance of adhering to the post op rehab plan is good practice |
| PO.16 *The specialist ward nurse/doctor* asks each patient whether they require a fit note on discharge  and completes the fit note in accordance with best practice guidelines and the hospital contract, and with reference to the patient’s RTW plan in their workbook | *The specialist ward nurse/doctor* describes the process of asking each patient whether they require a fit note on discharge   - How - When - Where   *The specialist ward nurse/doctor* describes the process of completing the fit note in accordance with best practice guidelines and the hospital contract, and with reference to the patient’s RTW plan in their workbook   - How - When - Where | *The specialist ward nurse/doctor* express confidence in their ability to ask each patient whether they require a fit note on discharge  *The specialist ward nurse/doctor* express confidence in their ability to complete the fit note in accordance with best practice guidelines and the hospital contract, and with reference to the patient’s RTW plan in their workbook | *The specialist ward nurse/doctor* state that asking each patient whether they require a fit note on discharge and completing the fit note in accordance with best practice guidelines and the hospital contract, and with reference to the patient’s RTW plan in their workbook will facilitate the patient’s RTW | *The specialist ward nurse/doctor* recognise that asking each patient whether they require a fit note on discharge and completing the fit note in accordance with best practice guidelines and the hospital contract, and with reference to the patient’s RTW plan is good practice |
| PO.17 *The RTWC* checks the RTW helpline 3 x wk, and triages, advises (e.g. phone call) or refers back to therapy services (based on local service structure and availability) based on individual need. | The RTWC describes the process of checking the helpline and the actions they are required to follow in response to the patient   - When - What - How | The RTWC expresses confidence in their ability to check the helpline and in taking the actions they are required to follow in response to the patient | The RTWC states that checking the helpline and taking the actions they are required to follow in response to the patient  will facilitate the patient’s RTW | The RTWC recognises that checking the helpline and taking the actions they are required to follow in response to the patient is good practice |
| PO.18 *Surgeon, HOT and outpatient therapy teams* summarise and record patient’s RTW status / outcome in all outpatient clinic notes and following each appointment | The surgeon, HOT and outpatient therapy teams describe the process of  summarising and recording patient’s RTW status / outcome in all outpatient clinic notes and following each appointment   - What - Where - How | The surgeon, HOT and outpatient therapy teams express confidence in their ability to  summarise and record patient’s RTW status / outcome in all outpatient clinic notes and following each appointment | The surgeon, HOT and outpatient therapy teams state that summarising and recording patient’s RTW status / outcome in all outpatient clinic notes and following each appointment will facilitate the patient’s RTW | The surgeon, HOT and outpatient therapy teams recognise that summarising and recording patient’s RTW status / outcome in all outpatient clinic notes and following each appointment is good practice |
| PO.19 *Surgeon and HOT* communicate with GP at point patient is discharged from orthopaedic surgical care, outlining current RTW status and progress and on-going therapy received and encourage engagement with RTWC until16 weeks post-surgery (8 weeks for feasibility study) | Surgeon and HOT describe the process of communicating with the GP at the point that the patient is discharged from orthopaedic surgical care, outlining current RTW status and progress and on-going therapy received | Surgeon and HOT express confidence in their ability to communicate with the GP at the point that the patient is discharged from orthopaedic surgical care, outlining current RTW status and progress and on-going therapy received | Surgeon and HOT state that communicating with the GP at the point that the patient is discharged from orthopaedic surgical care, outlining current RTW status and progress and on-going therapy received will facilitate the patient’s RTW | Surgeon and HOT state that communicating with the GP at the point that the patient is discharged from orthopaedic surgical care, outlining current RTW status and progress and on-going therapy received is good practice |
| PO.20 *RTWC* continues to provide a point of access to RTW advice for patients following discharge from orthopaedic surgical care until 16 weeks post-surgery (8 weeks for feasibility study)  Records any changes to patient’s RTW progress/status/outcome in RTWC workbook | *RTWC* describes the process of providing a point of access to RTW advice for patients following discharge from orthopaedic surgical care until 16 weeks post-surgery (8 weeks for feasibility study)  Describes the process of recording changes to patient’s RTW progress/status/outcome in RTWC workbook | *RTWC* expresses confidence in their ability to provide a point of access to RTW advice for patients following discharge from orthopaedic surgical care until 16 weeks post-surgery (8 weeks for feasibility study)  Expresses confidence in recording changes to patient’s RTW progress/status/outcome in RTWC workbook | *RTWC* state that providing a point of access to RTW advice for patients following discharge from orthopaedic surgical care until 16 weeks post-surgery (8 weeks for feasibility study)  will facilitate the patient’s RTW  States that recording changes to patient’s RTW progress/status/outcome in RTWC workbook will facilitate the patient's RTW | *RTWC* recognises that providing a point of access to RTW advice for patients following discharge from orthopaedic surgical care until 16 weeks post-surgery (8 weeks for feasibility study) is good practice  Recognises that recording changes to patient’s RTW progress/status/outcome in RTWC workbook is good practice |
